# The genus *Eriastichus* La Salle (Hymenoptera, Eulophidae, Tetrastichinae) in the Neotropical region, introducing 48 new species

**DOI:** 10.3897/zookeys.1019.60364

**Published:** 2021-02-22

**Authors:** Christer Hansson

**Affiliations:** 1 Scientific Associate Biological Museum (Entomology), Lund University, Sölvegatan 37, SE-22362 Lund, Sweden Lund University Lund Sweden; 2 Natural History Museum, Life Sciences, Cromwell Road, London, UK Natural History Museum London United Kingdom

**Keywords:** Autapomorphies, Costa Rica, identification key

## Abstract

Our knowledge of the recently described genus *Eriastichus* La Salle is greatly enhanced with the addition of 48 new species: *E. acribis***sp. nov.**, *E. aphritis***sp. nov.**, *E. cluridis***sp. nov.**, *E. coelotis***sp. nov.**, *E. colenis***sp. nov.**, *E. copalensis***sp. nov.**, *E. daptilis***sp. nov.**, *E. decoris***sp. nov.**, *E. denotatis***sp. nov.**, *E. derilis***sp. nov.**, *E. diadrys***sp. nov.**, *E. dotaensis***sp. nov.**, *E. drupis***sp. nov.**, *E. ebulis***sp. nov.**, *E. egrestis***sp. nov.**, *E. eleagnis***sp. nov.**, *E. ellipsis***sp. nov.**, *E. eminis***sp. nov.**, *E. facilis***sp. nov.**, *E. fenestris***sp. nov.**, *E. follis***sp. nov.**, *E. galeatis***sp. nov.**, *E. geratis***sp. nov.**, *E. glanis***sp. nov.**, *E. hilaris***sp. nov.**, *E. johnlasallei***sp. nov.**, *E. johnnoyesi***sp. nov.**, *E. maniatis***sp. nov.**, *E. nebulis***sp. nov.**, *E. neonis***sp. nov.**, *E. nexilis***sp. nov.**, *E. novalis***sp. nov.**, *E. nugalis***sp. nov.**, *E. oasis***sp. nov.**, *E. ononis***sp. nov.**, *E. orestis***sp. nov.**, *E. pallidops***sp. nov.**, *E. parabilis***sp. nov.**, *E. renodis***sp. nov.**, *E. rivalis***sp. nov.**, *E. sannionis***sp. nov.**, *E. scalaris***sp. nov.**, *E. sodalis***sp. nov.**, *E. taraxis***sp. nov.**, *E. tendrilis***sp. nov.**, *E. tonioazofeifai***sp. nov.**, *E. velaminis***sp. nov.**, and *E. vestis***sp. nov.** All species are known only from males, and all material is from Costa Rica. Females show little morphological variation and are not possible to separate to species or link to conspecific males at present. Apart from the diagnostic features for the genus presented in the original description, two new autapomorphies are introduced here: an inflated pleural membrane between Gt_1-4_ and Gs_1-4,_ and tufts of pale and flattened setae laterally on Gt_4-6_. Both features are found on the gaster and are present in both sexes. The distribution of the genus is firmly established as predominantly neotropical. The biology remains unknown.

## Introduction

Taxonomic knowledge of the Tetrastichinae (Chalcidoidea: Eulophidae) fauna in the Neotropical region is very poor, at both the genus and species levels. LaSalle et al. (2006) listed 29 genera in the subfamily from the Neotropical region, and with the addition of a new genus by Hansson (2020) this number is now 30, but hardly any of them have been investigated in this part of the world.

*Eriastichus* is a genus confined to the Americas, with the great majority of species occurring in tropical parts (La Salle 1994 and present findings). It was described in a paper on the Nearctic genera of Tetrastichinae (LaSalle 1994), including three species that were described in same paper. The species were based on females, and the male was included for only one of the species. With the exception of one specimen from the U.S.A. (Texas), the specimens were from the Neotropical region, ranging from the Caribbean (Dominican Republic) through Central America (Costa Rica, Mexico) to South America (Ecuador). The biology of the species was cited as being unknown.

## Materials and methods

### Material

Material forming the base of this study includes 63 females and 81 males, and all specimens were collected with a sweep net. The specimens were killed and kept in 80% ethanol until dried with a critical point drier. Thus, the specimens did not shrivel when dried and this should be kept in mind for the measurements/ratios given.

The types are deposited in the following museums with registration numbers prefixed by their acronyms:

**MZLU**Biological Museum (Entomology), Lund University, Lund, Sweden;

**MZUCR**Museo de Zoología, Universidad de Costa Rica, San José, Costa Rica;

**NHMUK**Natural History Museum, London, United Kingdom.

**Other museum acronyms**

**CNC**Canadian National Collection of Insects, Ottawa, Canada;

**USNM**United States National Museum of Natural History, Washington, D.C., USA.

### Imaging

The colour images of the specimens were made using Canon camera equipment including an EOS 5D Mark IV body, a telezoom lens, 70–300 mm (but using only 135 & 200 mm), with a 10 × Mitutoyo microscope lens attached, and macro twin lite MT-24 EX for illumination. The camera was attached to a Cognisys stackshot macrorail system. The picture stacking was done with Helicon Focus version 6, and Adobe Photoshop was used for image processing. The SEM micrographs are from uncoated specimens and were done with a Hitachi SU 3500 microscope, in low vacuum and using a backscatter detector.

### Morphological terminology

The terminology used here follows Gibson (1997), except the term mesoscutellum which is used instead of scutellum.

**Abbreviations of morphological terms**

**CC** costal cell in fore wing;

**F1–4** flagellomeres 1–4;

**Gs_1-7_** gastral sternites 1–7;

**Gt_1-7_** gastral tergites 1–7;

**HE** height of eye;

**MS** malar space;

**MV** marginal vein;

**OOL** shortest distance between lateral ocelli and eyes;

**PM** postmarginal vein in fore wing;

**POL** shortest distance between lateral ocelli;

**SLG** sublateral groove on mesoscutellum (sublateral line in LaSalle 1994);

**SMG** submedian groove on mesoscutellum (submedian line in LaSalle 1994);

**ST** stigmal vein in fore wing;

**WM** width of mouth opening.

### Species names

To avoid the problems associated with the requirement by the International Code of Zoological Nomenclature (International Commission of Zoological Nomenclature 1999) that the gender of species names must agree with that of the genus, all new names proposed in the present study, except when indicated otherwise, must be treated as arbitrary combinations of letters or nouns in apposition. As a consequence, species names will not change if species are transferred to a genus with a different gender at any time in the future.

## Taxonomic accounts

### Genus Eriastichus

Taxon classificationAnimaliaHymenopteraEulophidae

La Salle

EE2BFD3E-B985-5D62-8B6A-D3C290344CDA

La Salle 1994: 173–174. Type species Eriastichus cigdemae La Salle.

#### Diagnosis.

Heavily setose species (Figs 1, 2, 5, 6): head (including eyes), pronotum, mesoscutum, coxae, and lateral parts of propodeum (excl. bare median part – hairy only in *E. masneri* La Salle) ± covered with fine, short setae; malar sulcus curved; base of scape oblique (e.g., Fig. 45); F1–F4 of male antenna with scattered setae of varying length (Figs 11–58), i.e., without a compact whorl of long setae attached close to base – a feature present in many tetrastichine groups; mesoscutellum transverse (Figs 5–6); dorsellum large, flat to slightly convex and smooth (Fig. 5); fore wing with postmarginal vein 0.3–0.7 × as long as the elongate and slender stigmal vein, wing disc strongly setose, including base (i.e., without speculum) and ventral surface of costal cell (Fig. 4), wings ±uniformly but weakly infuscate (Fig. 4) but in a few species hyaline; gaster with pleural membrane between Gt_1-4_ and Gs_1-4_ inflated (Figs 1–3, 7, 8); both sexes with a tuft of pale and flattened setae laterally on Gt_3-6_ (Figs 7–10), though most species with tufts only on Gt_6_.

**Figures 1–6.**
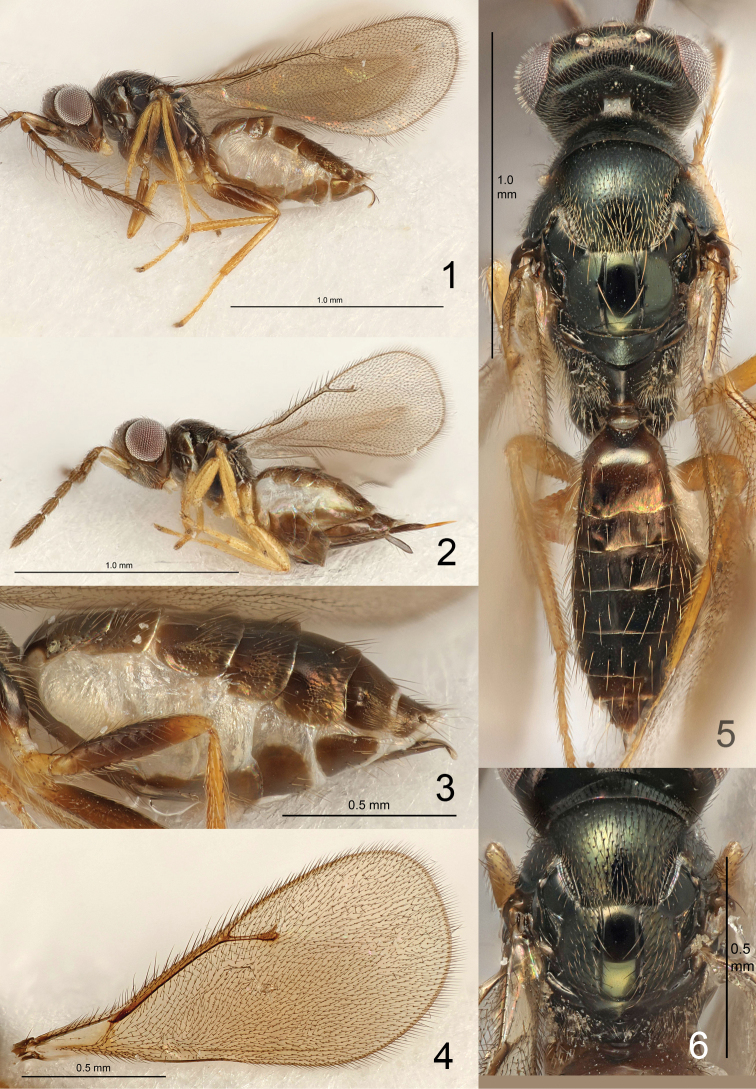
*Eriastichus* spp. **1***E. eleagnis*, body in lateral view, male paratype **2***E.* sp., body in lateral view, female **3***E. decoris*, gaster in lateral view, male holotype **4***E.* sp., right fore wing, female **5***E. fenestris*, body in dorsal view, male holotype **6***E.* sp., thoracic dorsum, female.

**Figures 7–10.**
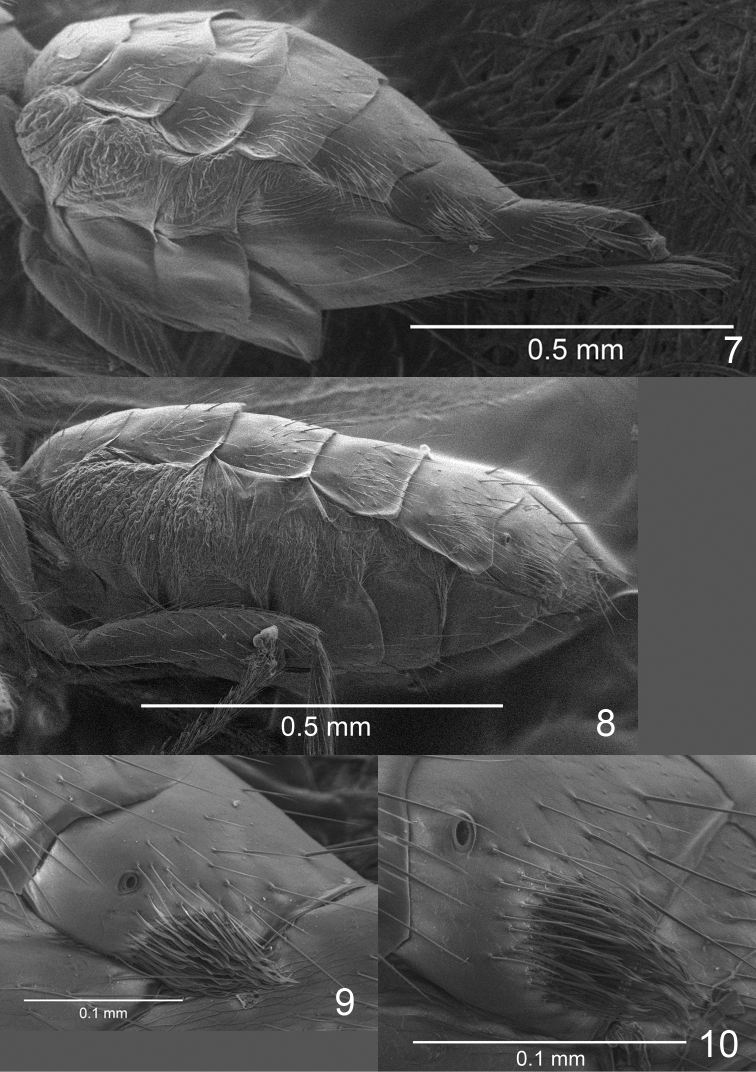
*Eriastichus* spp. **7, 8** gaster in lateral view: **7***E.* sp., female **8***E. ebulis*, male paratype **9, 10** Gt_6_ in lateral view, with brush of flattened setae: **9***E.* sp., female **10***E. ebulis*, male paratype.

##### Key to males

**Table:** 

1	Mesoscutellum with parts lateral to submedian grooves strongly setose (Fig. 6)	**2**
–	Mesoscutellum with parts lateral to submedian grooves with two or three pairs of strong setae (Fig. 5)	**3**
2	Median propodeum hairy (La Salle 1994: fig. 30)	***E. masneri* La Salle** (male unknown, this is an anticipated placement; not recorded from Costa Rica)
–	Median propodeum bare (as in Fig. 5)	***E. nakos* La Salle** (male unknown, this is an anticipated placement; not recorded from Costa Rica)
3	Scape 1.3–1.4 × as long as ventral plaque (Fig. 50); lower face yellowish brown (Fig. 59)	***E. pallidops* sp. nov.**
–	Scape 1.7–8.0 × as long as ventral plaque; colour of lower face variable	**4**
4	Scape 5.3–8.0 × as long as ventral plaque	**5**
–	Scape 1.7–5.0 × as long as ventral plaque	**9**
5	Antenna with dorsobasal setae on F1 0.5 × as long as length of F1 (Fig. 15), pedicel + flagellum 2.0 × as long as width of head	***E. copalensis* sp. nov.**
–	Antenna with dorsobasal setae on F1 at least 1.1 × as long as length of F1, pedicel + flagellum at most 1.6 × as long as width of head	**6**
6	Scape 8.0 × as long as ventral plaque (Fig. 57), and 1.4 × as long as height of eye	***E. tonioazofeifai* sp. nov.**
–	Scape at most 6.7 × as long as ventral plaque, and at most 0.9 × as long as height of eye	**7**
7	Antennal flagellum longer (Fig. 11), e.g., F1 2.5 ×, F4 2.8 ×, clava 7.3 × as long as wide	***E. acribis* sp. nov.**
–	Antennal flagellum shorter, F1 1.5–1.7 ×, F4 2.0 ×, clava 4.8–5.2 × as long as wide	**8**
8	Setae on flagellomeres more erect, and dorsobasal setae on F1 1.5 × as long as length of F1 (Fig. 12)	***E. cluridis* sp. nov.**
–	Setae on flagellomeres less erect, and dorsobasal setae on F1 1.1 × as long as length of F1 (Fig. 16)	***E. coelotis* sp. nov.**
9	Scape 1.3 × as long as height of eye (Fig. 14)	***E. colenis* sp. nov.**
–	Scape at most 1.0 × as long as height of eye	**10**
10	Scape dark brown	**11**
–	Scape yellowish brown to pale brown	**17**
11	Dorsobasal setae on F1 0.8–1.1 × as long as length of F1	**12**
–	Dorsobasal setae on F1 0.4–0.6 × as long as length of F1	**14**
12	Ventral plaque on scape situated medially (Fig. 37)	***E. johnlasallei* sp. nov.**
–	Ventral plaque on scape situated below the middle (Figs 17, 20)	**13**
13	Each flagellomere with few setae, e.g., F1 with six setae, ventral plaque 0.2 × as long as length of scape (Fig. 17)	***E. daptilis* sp. nov.**
–	Each flagellomere with more setae, e.g., F1 with ca. 15 setae, ventral plaque 0.4 × as long as length of scape (Fig. 20)	***E. dotaensis* sp. nov.**
14	Flagellomeres longer, F1, F3, F4 each 3.7 ×, F2 4.0 × as long as wide (Fig. 18)	***E. decoris* sp. nov.**
–	Flagellomeres shorter, F1 2.0–3.5, F2 2.4–3.5, F3 2.1–3.4, F4 2.3–3.4 × as long as wide	**15**
15	Fore and mid legs with coxae and femora yellowish brown	***E. denotatis* sp. nov.**
–	Fore and mid legs with coxae and femora dark brown	**16**
16	Ventral plaque 0.5 × as long as length of scape (Fig. 22)	***E. diadrys* sp. nov.**
–	Ventral plaque 0.3 × as long as length of scape (Fig. 23)	***E. ebulis* sp. nov.**
17	Dorsobasal setae on F1 ca. 2 × as long as F1	***E. cigdemae* La Salle**
–	Dorsobasal setae on F1 at most 1.5 × as long as F1	**18**
18	Dorsobasal setae on F1 at least 1.2 × as long as F1, and setae on flagellomeres erect (Figs 24, 27)	**19**
–	Dorsobasal setae on F1 at most 1.0 × as long as F1, setae on flagellomeres variable	**20**
19	Antenna shorter (Fig. 24), pedicel + flagellum 1.1 × as long as width of head	***E. egrestis* sp. nov.**
–	Antenna longer (Fig. 27), pedicel + flagellum 1.6–1.7 × as long as width of head	***E. eleagnis* sp. nov.**
20	Scape 4.5–5.0 × as long as ventral plaque	**21**
–	Scape 1.8–4.2 × as long as ventral plaque	**24**
21	Ventral plaque situated medially (Fig. 25)	***E. ellipsis* sp. nov.**
–	Ventral plaque situated below median part of scape	**22**
22	Dorsobasal setae on F1 0.6 × as long as length of F1 (Fig. 40)	***E. johnnoyesi* sp. nov.**
–	Dorsobasal setae on F1 0.9–1.0 × as long as length of F1 (Figs 28, 31)	**23**
23	Antenna with F1–F4 each 3.3–3.5 × as long as wide, setae on flagellomeres less erect (Fig. 31)	***E. eminis* sp. nov.**
–	Antenna with F1–F4 1.8–2.5 × as long as wide, setae on flagellomeres more erect (Fig. 28)	***E. facilis* sp. nov.**
24	Scape 1.7–2.0 × as long as ventral plaque	**25**
–	Scape 2.2–4.2 × as long as ventral plaque	**28**
25	Setae on flagellomeres more erect, scape dark brown (Fig. 29)	***E. fenestris* sp. nov.**
–	Setae on flagellomeres less erect, scape yellowish brown to pale brown	**26**
26	Antenna with F1–F4 each 2.0 × and clava 4.3 × as long as wide (Fig. 21)	***E. derilis* sp. nov.**
–	Antenna with F1–F4 each 2.7–3.0 × and clava 5.5–6.3 × as long as wide (Figs 13, 30)	**27**
27	Flagellomeres with more setae, e.g., F2 with eight setae on dorsal surface (Fig. 13)	***E. aphritis* sp. nov.**
–	Flagellomeres with less setae, e.g., F2 with four setae on dorsal surface (Fig. 30)	***E. follis* sp. nov.**
28	Frons and legs yellowish brown	***E. sannionis* sp. nov.**
–	Frons partly to completely dark brown, legs yellowish brown to dark brown	**29**
29	Coxae and femora predominantly to completely dark brown	**30**
–	Coxae and femora ±yellowish brown, some coxae or hind femur can be partly dark brown	**33**
30	Setae on flagellomeres erect (Figs 34, 58)	**31**
–	Setae on flagellomeres less erect than in alternate (Figs 33, 56)	**32**
31	Scape narrow at base (Fig. 34)	***E. galeatis* sp. nov.**
–	Scape wide at base (Fig. 58)	***E. vestis* sp. nov.**
32	Flagellomeres with few setae, e.g., F2 with three setae on dorsal surface (Fig. 56)	***E. velaminis* sp. nov.**
–	Flagellomeres with more setae, e.g., F2 with eight setae on dorsal surface (Fig. 33)	***E. geratis* sp. nov.**
33	Scape 3.5–4.2 × as long as ventral plaque	**34**
–	Scape 2.2–3.1 × as long as ventral plaque	**37**
34	Dorsobasal setae on F1 0.5–0.6 × as long as F1 (Figs 32, 36)	**35**
–	Dorsobasal setae on F1 0.8–1.0 × as long as F1 (Figs 39, 41)	**36**
35	Flagellomeres with more setae, e.g., F2 with eight setae on dorsal surface (Fig. 32)	***E. glanis* sp. nov.**
–	Flagellomeres with fewer setae, e.g., F2 with five setae on dorsal surface (Fig. 36)	***E. maniatis* sp. nov.**
36	Setae on flagellomeres more erect (Fig. 39)	***E. neonis* sp. nov.**
–	Setae on flagellomeres less erect (Fig. 41)	***E. nexilis* sp. nov.**
37	Ventral plaque situated below middle of scape	**38**
–	Ventral plaque situated in the middle, or slightly above the middle	**44**
38	Scape 2.2–2.5 × as long as ventral plaque	**39**
–	Scape 2.8–3.1 × as long as ventral plaque	**40**
39	Dorsobasal setae on F1 1.0 × as long as F1 (Fig. 44)	***E. novalis* sp. nov.**
–	Dorsobasal setae on F1 0.5–0.6 × as long as F1 (Fig. 42)	***E. oasis* sp. nov.**
40	F2–F4 each 2.3 × as long as wide (Fig. 26)	***E. drupis* sp. nov.**
–	F2–F4 each at least 3.0 × as long as wide	**41**
41	Flagellomeres longer, e.g., F2 3.6–3.8 × as long as wide (Figs 38, 43)	**42**
–	Flagellomeres shorter, F2 3.0–3.2 × as long as wide	**43**
42	Dorsobasal setae on F1 0.9 × as long as F1 (Fig. 43)	***E. ononis* sp. nov.**
–	Dorsobasal setae on F1 0.6 × as long as F1 (Fig. 38)	***E. nebulis* sp. nov.**
43	Dorsobasal setae on F1 0.5 × as long as F1, scape dark brown (Fig. 46)	***E. parabilis* sp. nov.**
–	Dorsobasal setae on F1 0.9 × as long as F1, scape yellowish brown (Fig. 49)	***E. orestis* sp. nov.**
44	Dorsobasal setae on F1 0.5 × as long as F1 and pedicel + flagellum 1.9 × as long as width of head	**45**
–	Dorsobasal setae on F1 at least 0.6 × as long as F1 and/or pedicel + flagellum at most 1.7 × as long as width of head	**46**
45	Setae on flagellomeres more erect (Fig. 47)	***E. rivalis* sp. nov.**
–	Setae on flagellomeres less erect (Fig. 45)	***E. nugalis* sp. nov.**
46	Dorsobasal setae on F1 0.4 × as long as F1 (Fig. 35) and pedicel + flagellum 1.7 × as long as width of head	***E. hilaris* sp. nov.**
–	Dorsobasal setae on F1 0.6–1.0 × as long as F1, if dorsobasal setae on F1 is only 0.6 × as long as F1, then pedicel + flagellum are 1.5 × as long as width of head	**47**
47	Antennal flagellum long and slender (Fig. 54), e.g., F4 3.4 × and clava 7.6 × as long as wide	***E. renodis* sp. nov.**
–	F4 2.2–2.8 × and clava 4.8–6.3 × as long as wide	**48**
48	Dorsobasal setae on F1–F4 1.0 × as long as funicular segment it is attached to (Fig. 52)	***E. scalaris* sp. nov.**
–	Dorsobasal setae on F1–F4 0.6–0.8 × as long as funicular segment it is attached to	**49**
49	Antennal flagellum more slender (Fig. 53), e.g., F3 3.0 × and clava 6.3 × as long as wide	***E. taraxis* sp. nov.**
–	F3 2.3–2.6 × and clava 4.8–5.8 × as long as wide	**50**
50	Pedicel + flagellum 1.9 × as long as width of head	***E. sodalis* sp. nov.**
–	Pedicel + flagellum 1.5–1.7 × as long as width of head	***E. tendrilis* sp. nov.**

##### Species treatments

### Eriastichus acribis sp. nov.

Taxon classificationAnimaliaHymenopteraEulophidae

1C39B4B3-15E2-5F3B-BD62-8540B3FF361C

http://zoobank.org/4CAF1EDF-8C16-4075-B08B-1356397DEC4B

Figure 11

#### Type locality.

Costa Rica, Heredia, Estación Biológica La Selva, 75 m, 10°26'N, 84°01'W, 24–25.ii.2011, J.S. Noyes leg.

#### Type specimen.

***Holotype*** male dried and glued to a paper card. Original labels: ”COSTA RICA, Heredia, E.B. La Selva, 75 m, 10°26'N, 84°01'W, 24–25.ii.2011, J.S. Noyes, NHM (Ent) 2011–93”, “HOLOTYPE Eriastichus acribis Hansson” [red printed label], (NHMUK014431021).

#### Diagnosis

**Figures 11–17.**
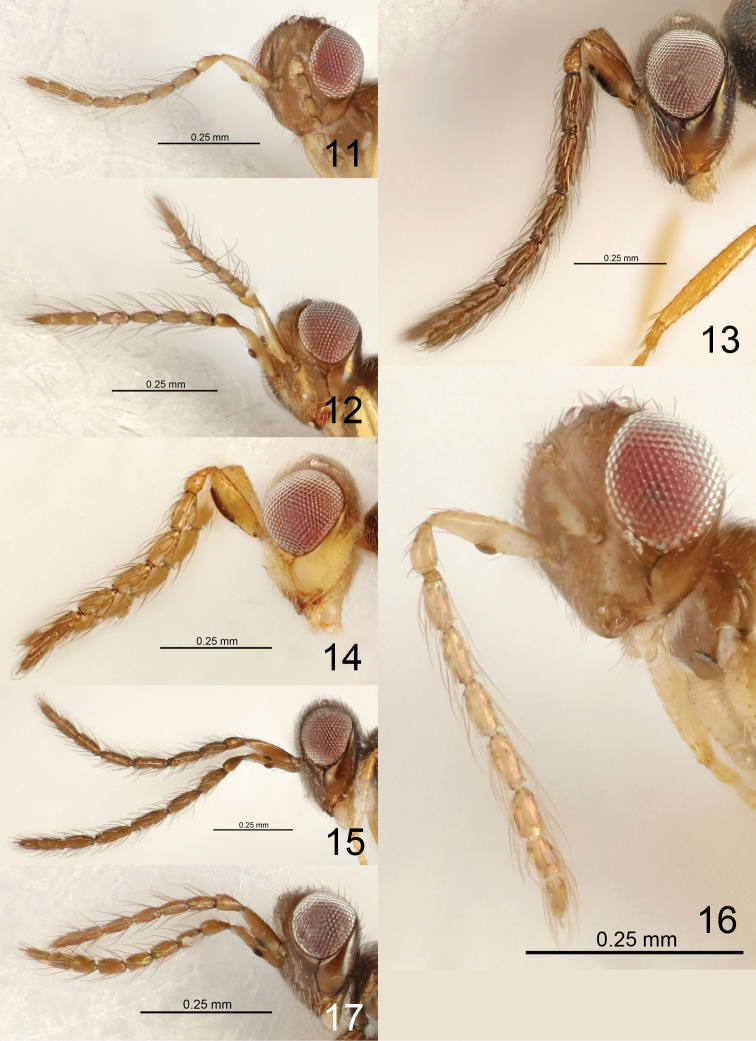
*Eriastichus* spp. head and antenna in lateral view, male holotypes **11***E. acribis***12***E. cluridis***13***E. aphritis***14***E. colenis***15***E. copalensis***16***E. coelotis***17***E. daptilis*.

**(male).** Head brown; antenna (Fig. 11): scape yellowish brown, ventral plaque on scape ca. 0.1 × as long as scape, antenna with dorsobasal setae on F1 1.1 × as long as F1; gaster with lateral tufts of pale and flattened setae on Gt_6_.

#### Description

**(male holotype NHMUK014431021).** Length of body 1.2 mm. Head brown. Antenna with scape yellowish brown, ventral plaque brown, pedicel and flagellum brown. Mesoscutum and mesoscutellum dark brown; dorsellum and propodeum pale brown. Legs yellowish brown. Gaster dark brown.

***Head*.** Length/width in frontal view 0.8; width/length in dorsal view 2.5; POL/OOL 2.8; WM/MS 3.6; MS/HE 0.4; HE/head length in frontal view 0.6; widths head/mesoscutum 1.2. ***Antenna*.** Pedicel + flagellum length/mesoscutum width 1.8; pedicel + flagellum length/head width 1.5; lengths scape/ventral plaque 6.7; ventral plaque located in the middle of scape; scape length/width 2.9; lengths scape/head (dorsal view) 0.5; scape length/HE 0.8; length/width F1, F2, F3, F4, clava: 2.5, 2.0, 2.3, 2.8, 7.3; length dorsobasal setae on F1/length F1 1.1. ***Mesosoma*.** Length/width 1.4; mesoscutum length/width 0.5; mesoscutellum length/width 0.6; widths SMG/SLG 1.0; enclosed space between SMG length/width 2.0; lengths mesoscutum/mesoscutellum 1.2; lengths mesoscutellum/dorsellum 2.3; lengths mesosoma/gaster 0.7. ***Wings*.**CC length/width 21.9; lengths CC/MV 1.0; lengths MV/ST 2.3; lengths MV/PM 4.9; lengths PM/ST 0.5; submarginal vein with four setae on dorsal surface. ***Gaster*.** With lateral tufts of pale and flattened setae on Gt_6_.

### Eriastichus aphritis sp. nov.

Taxon classificationAnimaliaHymenopteraEulophidae

9FE36D4D-B530-5FA9-8B31-AA4A77CF0314

http://zoobank.org/B8A47821-A1EE-4B64-B7C7-5EEE1B059956

Figure 13

#### Type locality.

Costa Rica, Puntarenas, San Vito, Estación Biológica Las Alturas, 1500 m, 8°57'N, 82°50'W, 17–18.ii.2012, J.S. Noyes leg.

#### Type specimen.

***Holotype*** male dried and glued to a paper card. Original labels: ”COSTA RICA, Puntarenas, San Vito, E.B. Las Alturas, 1500 m, 8°57'N, 82°50'W, 17–18.ii.2012, J.S. Noyes, NHM (Ent) 2012–91”, “HOLOTYPE Eriastichus aphritis Hansson” [red printed label], (NHMUK014431022).

#### Diagnosis

**(male).** Head black with metallic tinges with part below antennal toruli yellowish brown, scape and pedicel pale brown, flagellum brown; ventral plaque on scape ca. 0.5 × as long as scape (Fig. 13), antenna with dorsobasal setae on F1 0.5 × as long as F1; gaster with lateral tufts of pale and flattened setae on Gt_6_.

#### Description

**(male holotype NHMUK014431022).** Length of body 2.2 mm. Head black with metallic tinges with part below antennal toruli yellowish brown, scape and pedicel pale brown, flagellum brown, ventral plaque dark brown. Mesoscutum and mesoscutellum black with metallic tinges, dorsellum and propodeum dark brown. Legs yellowish brown. Gaster dark brown.

***Head*.** Length/width in frontal view 0.8; width/length in dorsal view 2.5; POL/OOL 1.9; WM/MS 1.5; MS/HE 0.6; HE/head length in frontal view 0.5; widths head/mesoscutum 1.2. ***Antenna*.** Pedicel + flagellum length/mesoscutum width 1.9; pedicel + flagellum length/head width 1.8; lengths scape/ventral plaque 2.1; ventral plaque located below the middle of scape; scape length/width 3.0; lengths scape/head (dorsal view) 0.5; scape length/HE 0.9; length/width F1, F2, F3, F4, clava: 3.0, 2.9, 2.8, 2.8, 5.5; length dorsobasal setae on F1/length F1 0.5. ***Mesosoma*.** Length/width 1.6; mesoscutum length/width 0.6; mesoscutellum length/width 0.8; widths SMG/SLG 1.1; enclosed space between SMG length/width 2.3; lengths mesoscutum/mesoscutellum 1.5; lengths mesoscutellum/dorsellum 3.4; lengths mesosoma/gaster 0.7. ***Wings*.**CC length/width 17.5; lengths CC/MV 1.1; lengths MV/ST 2.1; lengths MV/PM 3.0; lengths PM/ST 0.7; submarginal vein with ten setae on dorsal surface. ***Gaster*.** With lateral tufts of pale and flattened setae on Gt_6_.

### Eriastichus cigdemae

Taxon classificationAnimaliaHymenopteraEulophidae

La Salle

5C7D0CCB-F076-563E-B5D4-5E0DAB3EC940

Eriastichus cigdemae La Salle, 1994: 207. Holotype female in USNM, not examined.

#### Diagnosis.

Mesoscutellum with 2–3 pairs of setae; male with dorsobasal setae on F1 ca. 2× as long as F1.

#### Description.

See La Salle (1994).

#### Distribution.

Costa Rica, Mexico, U.S.A. (Texas) (La Salle 1994).

### Eriastichus cluridis sp. nov.

Taxon classificationAnimaliaHymenopteraEulophidae

7ACBE18C-1309-5FF4-A1FD-15BE9222C54B

http://zoobank.org/0C4B7F59-E6A9-4F47-AC10-BA87174C2738

Figure 12

#### Type locality.

Costa Rica, Alajuela, Parque Nacional Volcan Arenal, 10°26'N, 84°43'W, 600 m, 26.ii.2013, J.S. Noyes leg.

#### Type specimen.

***Holotype*** male dried and glued to a paper card. Original labels: ”Costa Rica, Alajuela, P.N. Volcan Arenal, 10°26'N, 84°43'W, 600 m, 26.ii.2013, J.S. Noyes, NHM (Ent) 2013–145AQ”, “HOLOTYPE Eriastichus cluridis Hansson” [red printed label],(NHMUK014431023).

#### Diagnosis

**(male).** Head dark brown with part below antennal toruli yellowish brown; antenna (Fig. 12): scape yellowish brown, ventral plaque on scape ca. 0.2 × as long as scape, antenna with dorsobasal setae on F1 1.5 × as long as F1; gaster with lateral tufts of pale and flattened setae on Gt_6_.

#### Description

**(male holotype NHMUK014431023).** Length of body 1.2 mm. Head dark brown with part below antennal toruli yellowish brown. Antenna with scape yellowish brown, ventral plaque dark brown, pedicel and flagellum brown. Mesoscutum, mesoscutellum and propodeum dark brown; dorsellum pale brown. Legs yellowish brown, fore and mid coxae brown at base, hind coxa and hind femur brown. Gaster dark brown.

***Head*.** Length/width in frontal view 0.8; width/length in dorsal view 2.1; POL/OOL 2.7; WM/MS 1.5; MS/HE 0.5; HE/head length in frontal view 0.6; widths head/mesoscutum 1.1. ***Antenna*.** Pedicel + flagellum length/mesoscutum width 1.6; pedicel + flagellum length/head width 1.4; lengths scape/ventral plaque 5.3; ventral plaque located in the middle of scape; scape length/width 3.0; lengths scape/head (dorsal view) 0.5; scape length/HE 0.9; length/width F1, F2, F3, F4, clava: 1.7, 1.7, 2.0, 2.0, 5.2; length dorsobasal setae on F1/length F1 1.5. ***Mesosoma*.** Length/width 1.4; mesoscutum length/width 0.6; mesoscutellum length/width 0.7; widths SMG/SLG 1.2; enclosed space between SMG length/width 1.7; lengths mesoscutum/mesoscutellum 1.4; lengths mesoscutellum/dorsellum 2.7; lengths mesosoma/gaster 0.8. ***Wings*.**CC length/width 25.0; lengths CC/MV 1.3; lengths MV/ST 2.0; lengths MV/PM 3.0; lengths PM/ST 0.7; submarginal vein with five setae on dorsal surface. ***Gaster*.** With lateral tufts of pale and flattened setae on Gt_6_.

### Eriastichus coelotis sp. nov.

Taxon classificationAnimaliaHymenopteraEulophidae

61411D2F-D745-538E-9239-964FEAE5AD8F

http://zoobank.org/D18D5E27-7C12-44CC-BBBB-0D18240246CB

Figure 16

#### Type locality.

Costa Rica, Heredia, Estación Biológica La Selva, 75 m, 10°26'N, 84°01'W, 23–24.ii.2005, J.S. Noyes leg.

#### Type specimen.

***Holotype*** male dried and glued to a paper card. Original labels: ”COSTA RICA, Heredia, E.B. La Selva, 75 m, 10°26'N, 84°01'W, 23–24.ii.2005, J.S. Noyes”, “HOLOTYPE Eriastichus coelotis Hansson” [red printed label], (NHMUK014431024).

#### Diagnosis

**(male).** Head dark brown; antenna (Fig. 16): scape yellowish brown, ventral plaque on scape ca. 0.2 × as long as scape, antenna with dorsobasal setae on F1 1.1 × as long as F1; gaster with lateral tufts of pale and flattened setae on Gt_6_.

#### Description

**(male holotype NHMUK014431024).** Length 1.1 mm. Head dark brown. Antenna with scape yellowish brown, ventral plaque dark brown, pedicel and flagellum pale brown. Mesoscutum, mesoscutellum and propodeum dark brown; dorsellum pale brown. Legs yellowish brown with base of hind coxa brown. Gaster dark brown.

***Head*.** Length/width in frontal view 0.8; width/length in dorsal view 2.3; POL/OOL 2.7; WM/MS 1.5; MS/HE 0.4; HE/head length in frontal view 0.7; widths head/mesoscutum 1.2. ***Antenna*.** Pedicel + flagellum length/mesoscutum width 1.7; pedicel + flagellum length/head width 1.5; lengths scape/ventral plaque 5.5; ventral plaque located in the middle of scape; scape length/width 3.4; lengths scape/head (dorsal view) 0.6; scape length/HE 0.8; length/width F1, F2, F3, F4, clava: 1.5, 1.8, 1.8, 2.0, 4.8; length dorsobasal setae on F1/length F1 1.1. ***Mesosoma*.** Length/width 1.5; mesoscutum length/width 0.6; mesoscutellum length/width 0.6; widths SMG/SLG 1.1; enclosed space between SMG length/width 1.8; lengths mesoscutum/mesoscutellum 1.5; lengths mesoscutellum/dorsellum 2.3; lengths mesosoma/gaster 0.8. ***Wings*.**CC length/width 22.5; lengths CC/MV 1.2; lengths MV/ST 2.1; lengths MV/PM 5.0; lengths PM/ST 0.4; submarginal vein with five setae on dorsal surface. ***Gaster*.** With lateral tufts of pale and flattened setae on Gt_6_.

### Eriastichus colenis sp. nov.

Taxon classificationAnimaliaHymenopteraEulophidae

B7785F05-45B5-57F9-9BCE-7E06FC921DCC

http://zoobank.org/2FA90FB6-94AF-4ABE-B176-ED2970A9177D

Figure 14

#### Type locality.

Costa Rica, Cartago, Humo, El Copal, 9°47'N, 83°45'W, 1050–1250 m, 29.ii–6.iii.2008, C. Hansson leg.

#### Type specimen.

***Holotype*** male dried and glued to a paper card. Original labels: “Costa Rica, Cartago, Humo, El Copal, 9°47'N, 83°45'W, 1050–1250 m, 29.ii–6.iii.2008, C. Hansson”, “HOLOTYPE Eriastichus colenis Hansson” [red printed label], (MZLU:7032.1).

#### Diagnosis

**(male).** Head yellowish brown with vertex and upper ½ of occiput dark brown; antenna (Fig. 14): scape yellowish brown, ventral plaque on scape ca. 0.4 × as long as scape, antenna with dorsobasal setae on F1 0.5 × as long as F1; gaster with lateral tufts of pale and flattened setae on Gt_5-6_.

#### Description

**(male holotype MZLU:7032.1).** Length of body 1.6 mm. Head yellowish brown with vertex and upper ½ of occiput dark brown. Antenna with scape yellowish brown, ventral plaque dark brown, pedicel and flagellum pale brown. Mesoscutum and propodeum dark brown; mesoscutellum dark brown with metallic tinges; dorsellum pale brown. Legs yellowish brown. Gaster dark brown.

***Head*.** Length/width in frontal view 0.7; width/length in dorsal view 2.1; POL/OOL 1.9; WM/MS 1.5; MS/HE 0.6; HE/head length in frontal view 0.6; widths head/mesoscutum 1.1. ***Antenna*.** Pedicel + flagellum length/mesoscutum width 1.7; pedicel + flagellum length/head width 1.5; lengths scape/ventral plaque 2.7; ventral plaque located above the middle of scape; scape length/width 4.2; lengths scape/head (dorsal view) 0.8; scape length/HE 1.3; length/width F1, F2, F3, F4, clava: 2.5, 2.3, 2.2, 2.2, 5.0; length dorsobasal setae on F1/length F1 0.5. ***Mesosoma*.** Length/width 1.5; mesoscutum length/width 0.5; mesoscutellum length/width 0.8; widths SMG/SLG 1.3; enclosed space between SMG length/width 2.0; lengths mesoscutum/mesoscutellum 1.2; lengths mesoscutellum/dorsellum 3.3; lengths mesosoma/gaster 0.8. ***Wings*.**CC length/width 21.7; lengths CC/MV 1.1; lengths MV/ST 1.9; lengths MV/PM 4.8; lengths PM/ST 0.4; submarginal vein with eight setae on dorsal surface. ***Gaster*.** With lateral tufts of pale and flattened setae on Gt_5-6_.

### Eriastichus copalensis sp. nov.

Taxon classificationAnimaliaHymenopteraEulophidae

B8DD91C2-1BB2-5D88-982D-FB77FC644B69

http://zoobank.org/55B02464-2EB7-45F9-8862-78EBF38D0631

Figure 15

#### Type locality.

Costa Rica, Cartago, Humo, El Copal, 9°47'N, 83°45'W, 1050–1250 m, 29.ii–6.iii.2008, C. Hansson leg.

#### Type specimen.

***Holotype*** male dried and glued to a paper card. Original labels: ”Costa Rica, Cartago, Humo, El Copal, 9°47'N, 83°45'W, 1050–1250 m, 29.ii–6.iii.2008, C. Hansson”, “HOLOTYPE Eriastichus copalensis Hansson” [red printed label], (MZLU:7033.1)

#### Diagnosis

**(male).** Head dark brown; antenna (Fig. 15): scape brown, ventral plaque on scape ca. 0.2 × as long as scape, antenna with dorsobasal setae on F1 0.5 × as long as F1; gaster with lateral tufts of pale and flattened setae on Gt_6_.

#### Description

**(male holotype MZLU:7033.1).** Length of body 1.5 mm. Head dark brown. Antenna brown, ventral plaque dark brown. Mesoscutum, mesoscutellum and propodeum dark brown; dorsellum pale brown. Legs with coxae and femora dark brown, trochanters, tibiae and tarsi yellowish brown. Gaster dark brown.

***Head*.** Length/width in frontal view 0.8; width/length in dorsal view 2.1; POL/OOL 2.5; WM/MS 1.7; MS/HE 0.5; HE/head length in frontal view 0.6; widths head/mesoscutum 1.2. ***Antenna*.** Pedicel + flagellum length/mesoscutum width 2.2; pedicel + flagellum length/head width 2.0; lengths scape/ventral plaque 6.0; ventral plaque located slightly above the middle of scape; scape length/width 3.8; lengths scape/head (dorsal view) 0.5; scape length/HE 0.8; length/width F1, F2, F3, F4, clava: 3.0, 3.4, 3.8, 3.4, 8.0; length dorsobasal setae on F1/length F1 0.5. ***Mesosoma*.** Length/width 1.6; mesoscutum length/width 0.6; mesoscutellum length/width 0.8; widths SMG/SLG 1.2; enclosed space between SMG length/width 2.2; lengths mesoscutum/mesoscutellum 1.4; lengths mesoscutellum/dorsellum 3.0; lengths mesosoma/gaster 0.8. ***Wings*.**CC length/width 24.0; lengths CC/MV 1.1; lengths MV/ST 1.7; lengths MV/PM 3.1; lengths PM/ST 0.5; submarginal vein with seven setae on dorsal surface. ***Gaster*.** With lateral tufts of pale and flattened setae on Gt_6_.

#### Etymology.

Named after collecting locality.

### Eriastichus daptilis sp. nov.

Taxon classificationAnimaliaHymenopteraEulophidae

BA441156-0E7E-5327-A20D-79F56BF79662

http://zoobank.org/C60FFDEF-AF6E-434F-AD51-8FA8464C025B

Figure 17

#### Type locality.

Costa Rica, Heredia, Estación Biológica La Selva, 75 m, 10°26'N, 84°01'W, 28–29.ii.2008, J.S. Noyes leg.

#### Type specimen.

***Holotype*** male dried and glued to a paper card. Original labels: ”COSTA RICA, Heredia, E.B. La Selva, 75 m, 10°26'N, 84°01'W, 28–29.ii.2008, J.S. Noyes, NHM (Ent) 2010–21”, “HOLOTYPE Eriastichus daptilis Hansson” [red printed label], (NHMUK014431025).

#### Diagnosis

**(male).** Head and antenna dark brown; ventral plaque on scape ca. 0.2 × as long as scape (Fig. 17), antenna with dorsobasal setae on F1 1.1 × as long as F1; dorsellum and major parts of legs dark brown; gaster without lateral tufts on tergites.

#### Description

**(male holotype NHMUK014431025).** Length of body 0.9 mm. Head and antenna dark brown, ventral plaque black. Mesoscutum, mesoscutellum, dorsellum and propodeum dark brown. Legs dark brown, trochanters and tarsi yellowish brown. Gaster dark brown.

***Head*.** Length/width in frontal view 0.8; width/length in dorsal view 2.1; POL/OOL 1.3; WM/MS 4.2; MS/HE 0.7; HE/head length in frontal view 0.6; widths head/mesoscutum 1.0. ***Antenna*.** Pedicel + flagellum length/mesoscutum width 1.6; pedicel + flagellum length/head width 1.5; lengths scape/ventral plaque 5.0; ventral plaque located below the middle of scape; scape length/width 3.6; lengths scape/head (dorsal view) 0.5; scape length/HE 0.9; length/width F1, F2, F3, F4, clava: 1.6, 1.8, 1.6, 1.6, 5.2; length dorsobasal setae on F1/length F1 1.1. ***Mesosoma*.** Length/width 1.3; mesoscutum length/width 0.5; mesoscutellum length/width 0.7; widths SMG/SLG 1.1; enclosed space between SMG length/width 1.9; lengths mesoscutum/mesoscutellum 1.3; lengths mesoscutellum/dorsellum 2.5; lengths mesosoma/gaster 0.9. ***Wings*.**CC length/width 16.3; lengths CC/MV 1.2; lengths MV/ST 1.7; lengths MV/PM 2.8; lengths PM/ST 0.6; submarginal vein with four setae on dorsal surface. ***Gaster*.** Without lateral tufts on tergites.

### Eriastichus decoris sp. nov.

Taxon classificationAnimaliaHymenopteraEulophidae

8C943A92-8927-5FA4-A10D-3D2BB4C56DBD

http://zoobank.org/A40C4518-95ED-4927-B6E7-5E3A49D80A33

Figures 3 , 18

#### Type locality.

Costa Rica, San José, San Gerardo de Dota, 9°33'N, 83°47'W, 20–21.ii.2013, J.S. Noyes leg.

#### Type specimen.

***Holotype*** male dried and glued to a paper card. Original labels: ”Costa Rica, San José, San Gerardo de Dota, 20–21.ii.2013, J.S. Noyes, NHM (Ent) 2012–91”, “HOLOTYPE Eriastichus decoris Hansson” [red printed label], (NHMUK014431026).

#### Diagnosis

**Figures 18–27.**
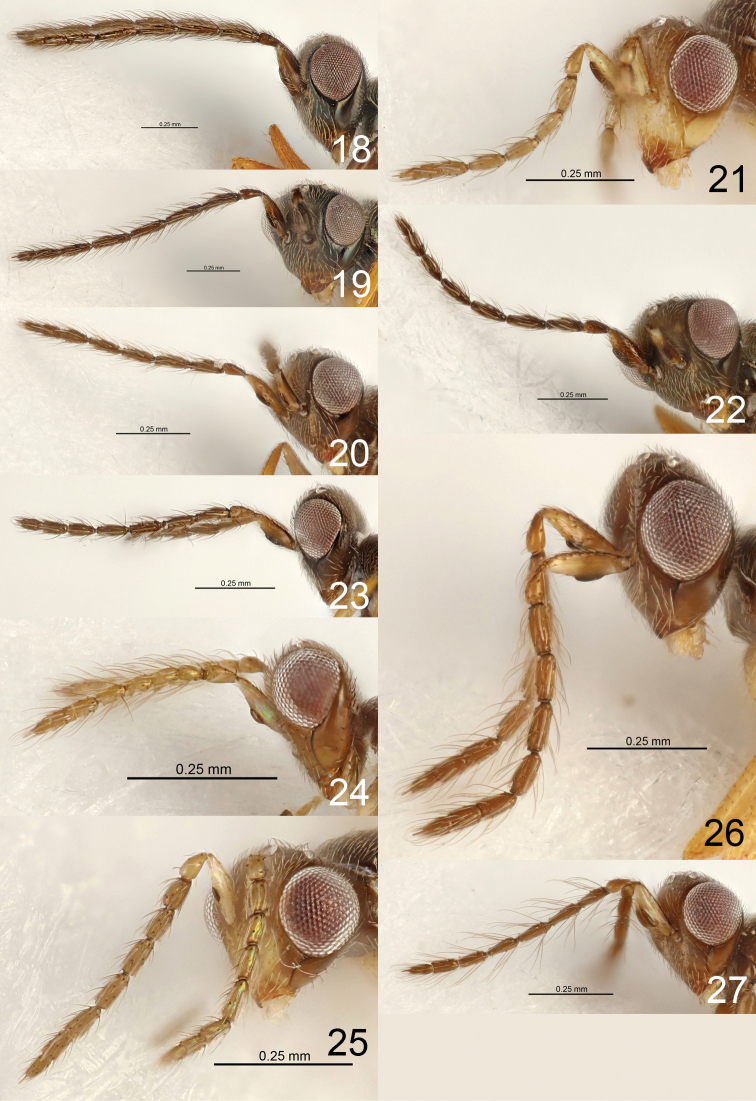
*Eriastichus* spp. head and antenna in lateral view, male holotypes **18***E. decoris***19***E. denotatis***20***E. dotaensis***21***E. derilis***22***E. diadrys***23***E. ebulis***24***E. egrestis***25***E. ellipsis***26***E. drupis***27***E. eleagnis*.

**(male).** Head black and antenna dark brown; ventral plaque on scape ca. 0.3 × as long as scape (Fig. 18), antenna with dorsobasal setae on F1 0.5 × as long as F1; gaster with lateral tufts of pale and flattened setae on Gt_5-6_.

#### Description

**(male holotype NHMUK014431026).** Length of body 2.3 mm. Head black, antenna dark brown, ventral plaque black. Mesoscutum and mesoscutellum black with metallic tinges, dorsellum and propodeum black. Legs with coxae, hind femur and 4^th^ tarsomere on all legs dark brown, fore femur pale brown, trochanters, tibiae, mid femur, and tarsomeres 1–3 yellowish brown. Gaster dark brown.

***Head*.** Length/width in frontal view 0.8; width/length in dorsal view 2.1; POL/OOL 2.0; WM/MS 1.5; MS/HE 0.6; HE/head length in frontal view 0.5; widths head/mesoscutum 1.2. ***Antenna*.** Pedicel + flagellum length/mesoscutum width 2.1; pedicel + flagellum length/head width 2.1; lengths scape/ventral plaque 3.1; ventral plaque located below the middle of scape; scape length/width 3.4; lengths scape/head (dorsal view) 0.5; scape length/HE 0.9; length/width F1, F2, F3, F4, clava: 3.7, 4.0, 3.7, 3.7, 7.9; length dorsobasal setae on F1/length F1 0.5. ***Mesosoma*.** Length/width 1.6; mesoscutum length/width 0.7; mesoscutellum length/width 0.8; widths SMG/SLG 1.1; enclosed space between SMG length/width 2.5; lengths mesoscutum/mesoscutellum 1.6; lengths mesoscutellum/dorsellum 2.6; lengths mesosoma/gaster 0.7. ***Wings*.**CC length/width 18.0; lengths CC/MV 0.9; lengths MV/ST 2.3; lengths MV/PM 3.8; lengths PM/ST 0.6; submarginal vein with nine setae on dorsal surface. ***Gaster*.** With lateral tufts of pale and flattened setae on Gt_5-6_.

### Eriastichus denotatis sp. nov.

Taxon classificationAnimaliaHymenopteraEulophidae

1943F1BC-B925-5EFA-86C1-366A66D96986

http://zoobank.org/306B5D9A-CBB9-447E-A4BD-57138BE907CD

Figures 19 , 60

#### Type locality.

Costa Rica, San José, San Gerardo de Dota, 9°33'N, 83°47'W, 20–21.ii.2013, J.S. Noyes leg.

#### Type specimen.

***Holotype*** male dried and glued to a paper card. Original labels: ”Costa Rica, San José, San Gerardo de Dota, 20–21.ii.2013, J.S. Noyes, NHM (Ent) 2012–91”, “HOLOTYPE Eriastichus denotatis Hansson” [red printed label], (NHMUK014431027).

#### Additional type material.

***Paratype*** 1♂ with same label data as holotype (NHMUK014431028).

#### Diagnosis

**(male).** Head black (Fig. 60) and antenna dark brown (Fig. 19); ventral plaque on scape ca. 0.4 × as long as scape (Fig. 19), antenna with dorsobasal setae on F1 0.6 × as long as F1; gaster with lateral tufts of pale and flattened setae on Gt_6_.

#### Description

**(male holotype NHMUK014431027).** Length of body 2.6 mm (paratype 2.3 mm). Head black, antenna dark brown, ventral plaque black. Mesoscutum and mesoscutellum black with metallic tinges, dorsellum and propodeum black. Legs with hind coxa dark brown, hind femur brown, fore and mid coxae and femora, trochanters, tibiae and tarsi yellowish brown. Gaster dark brown.

***Head*.** Length/width in frontal view 0.8; width/length in dorsal view 2.1; POL/OOL 1.6; WM/MS 1.5; MS/HE 0.6; HE/head length in frontal view 0.5; widths head/mesoscutum 1.1. ***Antenna*.** Pedicel + flagellum length/mesoscutum width 2.4; pedicel + flagellum length/head width 2.3; lengths scape/ventral plaque 2.4; ventral plaque located in the middle of scape; scape length/width 3.0; lengths scape/head (dorsal view) 0.5; scape length/HE 1.0; length/width F1, F2, F3, F4, clava: 3.1, 3.4, 3.4, 3.4, 7.3; length dorsobasal setae on F1/length F1 0.6. ***Mesosoma*.** Length/width 1.8; mesoscutum length/width 0.8; mesoscutellum length/width 0.9; widths SMG/SLG 1.2; enclosed space between SMG length/width 2.9; lengths mesoscutum/mesoscutellum 1.6; lengths mesoscutellum/dorsellum 2.7; lengths mesosoma/gaster 0.7. ***Wings*.**CC length/width 21.7; lengths CC/MV 0.9; lengths MV/ST 2.3; lengths MV/PM 3.9; lengths PM/ST 0.6; submarginal vein with nine setae (ten setae in paratype) on dorsal surface. ***Gaster*.** With lateral tufts of pale and flattened setae on Gt_6_.

### Eriastichus derilis sp. nov.

Taxon classificationAnimaliaHymenopteraEulophidae

239DF86C-6B4B-5897-BC1A-0EF5EA26FB87

http://zoobank.org/59EEBC97-92F5-43EB-9205-3816D5D672B0

Figure 21

#### Type locality.

Costa Rica, Cartago, Humo, El Copal, 9°47'N, 83°45'W, 1050–1250 m, 29.ii–6.iii.2008, C. Hansson leg.

#### Type specimen.

***Holotype*** male dried and glued to a paper card. Original labels: ”Costa Rica, Cartago, Humo, El Copal, 9°47'N, 83°45'W, 1050–1250 m, 29.ii–6.iii.2008, C. Hansson”, “HOLOTYPE Eriastichus derilis Hansson” [red printed label], (MZLU:7034.1).

#### Diagnosis

**(male).** Head with lower ½ yellowish brown and upper ½ dark brown, scape yellowish brown, pedicel and flagellum brown; ventral plaque on scape ca. 0.5 × as long as scape (Fig. 21), antenna with dorsobasal setae on F1 0.6 × as long as F1; gaster with lateral tufts of pale and flattened setae on Gt_5-6_.

#### Description

**(male holotype MZLU:7034.1).** Length of body 1.2 mm. Head with lower ½ yellowish brown and upper ½ dark brown, scape yellowish brown, pedicel and flagellum brown, ventral plaque dark brown. Mesoscutum, mesoscutellum, dorsellum and propodeum dark brown. Legs yellowish brown. Gaster dark brown.

***Head*.** Length/width in frontal view 0.8; width/length in dorsal view 2.3; POL/OOL 1.9; WM/MS 1.6; MS/HE 0.5; HE/head length in frontal view 0.6; widths head/mesoscutum 1.2. ***Antenna*.** Pedicel + flagellum length/mesoscutum width 1.7; pedicel + flagellum length/head width 1.5; lengths scape/ventral plaque 2.0; ventral plaque located above the middle of scape; scape length/width 3.0; lengths scape/head (dorsal view) 0.5; scape length/HE 0.9; length/width F1, F2, F3, F4, clava: 2.0, 2.0, 2.0, 2.0, 4.3; length dorsobasal setae on F1/length F1 0.6. ***Mesosoma*.** Length/width 1.4; mesoscutum length/width 0.6; mesoscutellum length/width 0.9; widths SMG/SLG 1.2; enclosed space between SMG length/width 2.2; lengths mesoscutum/mesoscutellum 1.3; lengths mesoscutellum/dorsellum 4.0; lengths mesosoma/gaster 0.8. ***Wings*.**CC length/width 21.0; lengths CC/MV 1.1; lengths MV/ST 1.8; lengths MV/PM 5.0; lengths PM/ST 0.4; submarginal vein with six setae on dorsal surface. ***Gaster*.** With lateral tufts of pale and flattened setae on Gt_5-6_.

### Eriastichus diadrys sp. nov.

Taxon classificationAnimaliaHymenopteraEulophidae

3772C259-4C26-5EC1-A868-0D37E227C121

http://zoobank.org/08F408C7-5C9C-4BFA-9A92-C11C2A11C881

Figure 22

#### Type locality.

Costa Rica, San José, San Gerardo de Dota, 9°33'N, 83°47'W, 20–21.ii.2013, J.S. Noyes leg.

#### Type specimen.

***Holotype*** male dried and glued to a paper card. Original labels: ”Costa Rica, San José, San Gerardo de Dota, 20–21.ii.2013, J.S. Noyes, NHM (Ent) 2012–91”, “HOLOTYPE Eriastichus diadrys Hansson” [red printed label], (NHMUK014431029).

#### Diagnosis

**(male).** Head black and antenna dark brown; ventral plaque on scape ca. 0.5 × as long as scape (Fig. 22), antenna with dorsobasal setae on F1 0.5 × as long as F1; gaster with lateral tufts of pale and flattened setae on Gt_3-6_.

#### Description

**(male holotype NHMUK014431029).** Length of body 2.1 mm. Head black, antenna dark brown, ventral plaque black. Mesoscutum and mesoscutellum black with metallic tinges, dorsellum and propodeum black. Legs with hind coxa and hind femur dark brown, fore and mid coxae yellowish brown with base dark brown, trochanters, tibiae and tarsi yellowish brown. Gaster dark brown.

***Head*.** Length/width in frontal view 0.7; width/length in dorsal view 2.2; POL/OOL 2.0; WM/MS 1.9; MS/HE 0.5; HE/head length in frontal view 0.6; widths head/mesoscutum 1.1. ***Antenna*.** Pedicel + flagellum length/mesoscutum width 1.9; pedicel + flagellum length/head width 1.8; lengths scape/ventral plaque 2.1; ventral plaque located in the middle of scape; scape length/width 2.7; lengths scape/head (dorsal view) 0.5; scape length/HE 0.9; length/width F1, F2, F3, F4, clava: 2.7, 2.9, 2.7, 2.6, 5.7; length dorsobasal setae on F1/length F1 0.5. ***Mesosoma*.** Length/width 1.6; mesoscutum length/width 0.7; mesoscutellum length/width 0.8; widths SMG/SLG 1.5; enclosed space between SMG length/width 2.0; lengths mesoscutum/mesoscutellum 1.6; lengths mesoscutellum/dorsellum 2.9; lengths mesosoma/gaster 0.8. ***Wings*.**CC length/width 20.6; lengths CC/MV 1.0; lengths MV/ST 2.1; lengths MV/PM 4.8; lengths PM/ST 0.4; submarginal vein with eight setae on dorsal surface. ***Gaster*.** With lateral tufts of pale and flattened setae on Gt_3-6_.

### Eriastichus dotaensis sp. nov.

Taxon classificationAnimaliaHymenopteraEulophidae

C9428DF8-0584-591E-8111-E82F9B846222

http://zoobank.org/E2228FB1-DE88-47A6-A504-CDEDE886BB50

Figure 20

#### Type locality.

Costa Rica, San José, San Gerardo de Dota, 9°33'N, 83°47'W, 20–21.ii.2013, J.S. Noyes leg.

#### Type specimen.

***Holotype*** male dried and glued to a paper card. Original labels: ”Costa Rica, San José, San Gerardo de Dota, 20–21.ii.2013, J.S. Noyes, NHM (Ent) 2012–91”, “HOLOTYPE Eriastichus dotaensis Hansson” [red printed label], (NHMUK014431030).

#### Diagnosis

**(male).** Head and antenna dark brown; ventral plaque on scape ca. 0.4 × as long as scape (Fig. 20), antenna with dorsobasal setae on F1 0.8 × as long as F1; gaster with lateral tufts of pale and flattened setae on Gt_6_.

#### Description

**(male holotype NHMUK014431030).** Length of body 1.6 mm. Head and antenna dark brown, ventral plaque black. Mesoscutum and mesoscutellum black with metallic tinges, dorsellum pale brown, propodeum dark brown. Legs with coxae and femora dark brown, trochanters, tibiae and tarsi yellowish brown. Gaster dark brown.

***Head*.** Length/width in frontal view 0.7; width/length in dorsal view 2.2; POL/OOL 1.7; WM/MS 1.5; MS/HE 0.6; HE/head length in frontal view 0.6; widths head/mesoscutum 1.2. ***Antenna*.** Pedicel + flagellum length/mesoscutum width 2.1; pedicel + flagellum length/head width 1.9; lengths scape/ventral plaque 2.7; ventral plaque located below the middle of scape; scape length/width 3.0; lengths scape/head (dorsal view) 0.6; scape length/HE 1.0; length/width F1, F2, F3, F4, clava: 2.5, 2.8, 2.7, 2.7, 6.4; length dorsobasal setae on F1/length F1 0.8. ***Mesosoma*.** Length/width 1.5; mesoscutum length/width 0.7; mesoscutellum length/width 0.8; widths SMG/SLG 1.1; enclosed space between SMG length/width 2.2; lengths mesoscutum/mesoscutellum 1.6; lengths mesoscutellum/dorsellum 2.6; lengths mesosoma/gaster 0.8. ***Wings*.**CC length/width 26.0; lengths CC/MV 1.0; lengths MV/ST 2.2; lengths MV/PM 3.6; lengths PM/ST 0.6; submarginal vein with six setae on dorsal surface. ***Gaster*.** With lateral tufts of pale and flattened setae on Gt_6_.

#### Etymology.

Named after collecting locality.

### Eriastichus drupis sp. nov.

Taxon classificationAnimaliaHymenopteraEulophidae

AED9991B-F8E7-518A-980C-59FD33842767

http://zoobank.org/4CF0E18A-2BE0-4E3F-BB19-378178073525

Figure 26

#### Type locality.

Costa Rica, Puntarenas, San Vito, Estación Biológica Las Alturas, 1500 m, 8°57'N, 82°50'W, 17–18.ii.2012, J.S. Noyes leg.

#### Type specimen.

***Holotype*** male dried and glued to a paper card. Original labels: ”COSTA RICA, Puntarenas, San Vito, E.B. Las Alturas, 1500 m, 8°57'N, 82°50'W, 17–18.ii.2012, J.S. Noyes, NHM (Ent) 2012–91”, “HOLOTYPE Eriastichus drupis Hansson” [red printed label], (NHMUK014431031).

#### Diagnosis

**(male).** Head dark brown, scape and pedicel pale brown, flagellum brown; ventral plaque on scape ca. 0.4 × as long as scape (Fig. 26), antenna with dorsobasal setae on F1 0.8 × as long as F1; gaster with lateral tufts of pale and flattened setae on Gt_5-6_.

#### Description

**(male holotype NHMUK014431031).** Length of body 1.4 mm. Head dark brown, scape and pedicel pale brown, flagellum brown, ventral plaque dark brown. Mesoscutum and mesoscutellum black with metallic tinges, dorsellum pale brown, propodeum dark brown. Legs with hind coxa and hind femur dark brown, fore and mid coxae yellowish brown with base brown, remaining parts of legs yellowish brown. Gaster dark brown.

***Head*.** Length/width in frontal view 0.8; width/length in dorsal view 2.2; POL/OOL 1.8; WM/MS 1.6; MS/HE 0.5; HE/head length in frontal view 0.6; widths head/mesoscutum 1.3. ***Antenna*.** Pedicel + flagellum length/mesoscutum width 1.7; pedicel + flagellum length/head width 1.6; lengths scape/ventral plaque 2.8; ventral plaque located below the middle of scape; scape length/width 3.1; lengths scape/head (dorsal view) 0.5; scape length/HE 0.9; length/width F1, F2, F3, F4, clava: 2.7, 2.3, 2.3, 2.2, 5.3; length dorsobasal setae on F1/length F1 0.8. ***Mesosoma*.** Length/width 1.5; mesoscutum length/width 0.5; mesoscutellum length/width 0.7; widths SMG/SLG 1.1; enclosed space between SMG length/width 2.1; lengths mesoscutum/mesoscutellum 1.3; lengths mesoscutellum/dorsellum 3.1; lengths mesosoma/gaster 0.9. ***Wings*.**CC length/width 22.5; lengths CC/MV 1.1; lengths MV/ST 1.9; lengths MV/PM 4.8; lengths PM/ST 0.4; submarginal vein with six setae on dorsal surface. ***Gaster*.** With lateral tufts of pale and flattened setae on Gt_5-6_.

### Eriastichus ebulis sp. nov.

Taxon classificationAnimaliaHymenopteraEulophidae

97B24A84-38A5-5DE6-A001-82BC660EC1E3

http://zoobank.org/44D3C6E8-F596-4AA4-BFD7-DCA4E8D2EC18

Figures 8 , 10 , 23

#### Type locality.

Costa Rica, San José, San Gerardo de Dota, 9°33'N, 83°47'W, 20–21.ii.2013, J.S. Noyes leg.

#### Type specimen.

***Holotype*** male dried and glued to a paper card. Original labels: ”Costa Rica, San José, San Gerardo de Dota, 20–21.ii.2013, J.S. Noyes, NHM (Ent) 2012–91”, “HOLOTYPE Eriastichus ebulis Hansson” [red printed label], (NHMUK014431032).

#### Additional type material.

***Paratypes*** (4♂♂): 2♂♂ with same label data as holotype (NHMUK014431033, NHMUK014431034); 2♂♂ “Costa Rica, Cartago, Humo, El Copal, 9°47'N, 83°45'W, 1050–1250 m, 29.ii–6.iii.2008, C. Hansson” (MZLU:7035.2, MZUCR:01693).

#### Diagnosis

**(male).** Head dark brown, antenna brown; ventral plaque on scape ca. 0.3 × as long as scape (Fig. 23), antenna with dorsobasal setae on F1 0.5 × as long as F1; gaster with lateral tufts of pale and flattened setae on Gt_5-6_.

#### Description

**(male holotype NHMUK014431032).** Length of body 1.5 mm (paratypes 1.6–2.0 mm). Head dark brown, antenna brown, ventral plaque black. Mesoscutum and mesoscutellum black with metallic tinges, dorsellum and propodeum black. Legs with coxae, femora and 4^th^ tarsomere on all legs dark brown, trochanters, tibiae and tarsomeres 1–4 yellowish brown. Gaster dark brown.

***Head*.** Length/width in frontal view 0.8; width/length in dorsal view 2.0; POL/OOL 1.5; WM/MS 1.4; MS/HE 0.6; HE/head length in frontal view 0.6; widths head/mesoscutum 1.0. ***Antenna*.** Pedicel + flagellum length/mesoscutum width 2.3; pedicel + flagellum length/head width 1.9; lengths scape/ventral plaque 3.0; ventral plaque located in the middle of scape; scape length/width 3.0; lengths scape/head (dorsal view) 0.5; scape length/HE 0.9; length/width F1, F2, F3, F4, clava: 3.2, 3.0, 3.0, 3.0, 7.8; length dorsobasal setae on F1/length F1 0.5. ***Mesosoma*.** Length/width 1.8; mesoscutum length/width 0.8; mesoscutellum length/width 0.8; widths SMG/SLG 1.6; enclosed space between SMG length/width 2.2; lengths mesoscutum/mesoscutellum 1.6; lengths mesoscutellum/dorsellum 2.7; lengths mesosoma/gaster 0.8. ***Wings*.**CC length/width 26.0; lengths CC/MV 1.0; lengths MV/ST 1.9; lengths MV/PM 7.4; lengths PM/ST 0.3; submarginal vein with seven setae (5–9 setae in paratypes) on dorsal surface. ***Gaster*.** With lateral tufts of pale and flattened setae on Gt_5-6_.

### Eriastichus egrestis sp. nov.

Taxon classificationAnimaliaHymenopteraEulophidae

92CBE1D6-AFD5-5263-8C82-FBFBF48A0DE1

http://zoobank.org/B875A5F8-F524-407E-8D2F-6A874BE758FA

Figure 24

#### Type locality.

Costa Rica, Puntarenas, San Vito, Las Cruces, 8°46'N, 82°57'W, 1300 m, 15–16.ii.2006, J.S. Noyes leg.

#### Type specimen.

***Holotype*** male dried and glued to a paper card. Original labels: ”COSTA RICA, Puntarenas, San Vito, Las Cruces, 8°46'N, 82°57'W, 1300 m, 15–16.ii.2006, J.S. Noyes”, “HOLOTYPE Eriastichus egrestis Hansson” [red printed label], (NHMUK014431035).

#### Diagnosis

**(male).** Head dark brown, antenna brown; ventral plaque on scape ca. 0.2 × as long as scape (Fig. 24), antenna with dorsobasal setae on F1 1.3 × as long as F1; gaster with lateral tufts of pale and flattened setae on Gt_6_.

#### Description

**(male holotype NHMUK014431035).** Length of body 1.0 mm. Head dark brown, antenna brown, ventral plaque dark brown. Mesoscutum and mesoscutellum dark brown with metallic tinges, dorsellum and propodeum dark brown. Legs with coxae and femora brown, trochanters, tibiae and tarsi yellowish brown. Gaster dark brown.

***Head*.** Length/width in frontal view 0.8; width/length in dorsal view 2.5; POL/OOL 2.4; WM/MS 1.5; MS/HE 0.6; HE/head length in frontal view 0.5; widths head/mesoscutum 1.3. ***Antenna*.** Pedicel + flagellum length/mesoscutum width 1.4; pedicel + flagellum length/head width 1.1; lengths scape/ventral plaque 4.5; ventral plaque located below the middle of scape; scape length/width 3.6; lengths scape/head (dorsal view) 0.5; scape length/HE 0.9; length/width F1, F2, F3, F4, clava: 1.4, 1.6, 1.6, 1.6, 4.3; length dorsobasal setae on F1/length F1 1.3. ***Mesosoma*.** Length/width 1.5; mesoscutum length/width 0.6; mesoscutellum length/width 0.6; widths SMG/SLG 1.4; enclosed space between SMG length/width 1.5; lengths mesoscutum/mesoscutellum 1.5; lengths mesoscutellum/dorsellum 2.5; lengths mesosoma/gaster 0.8. ***Wings*.**CC length/width 14.5; lengths costal cell/MV 1.0; lengths MV/ST 2.0; lengths MV/PM 3.5; lengths PM/ST 0.6; submarginal vein with five setae on dorsal surface. ***Gaster*.** With lateral tufts of pale and flattened setae on Gt_6_.

### Eriastichus eleagnis sp. nov.

Taxon classificationAnimaliaHymenopteraEulophidae

D9EAD11F-D460-5BDF-8F06-D3F7C8D093E8

http://zoobank.org/81F12226-5418-40DF-8BD8-D872B5B814B4

Figures 1 , 27

#### Type locality.

Costa Rica, Puntarenas, Estación Biológica Monteverde, 10°20'N, 84°49'W, 1540 m, 18–25.ii.2004, C. Hansson leg.

#### Type specimen.

***Holotype*** male dried and glued to a paper card. Original labels: ”COSTA RICA, Puntarenas, E.B. Monteverde, 10°20'N, 84°49'W, 1540 m, 18–25.ii.2004, C. Hansson”, “HOLOTYPE Eriastichus eleagnis Hansson” [red printed label], (MZLU:7036.1).

#### Additional type material.

***Paratypes*** (4♂♂): 1♂ with same label data as holotype (MZLU:7036.2); 1♂ from same locality as holotype but collected 26.ii.2007 (NHMUK014431036); 1♂ “COSTA RICA, Puntarenas, San Vito, E.B. Las Alturas, 1500 m, 8°57'N, 82°50'W, 17–18.ii.2012, J.S. Noyes, NHM (Ent) 2012–91” (NHMUK014431037); 1♂ “Costa Rica, San José, San Gerardo de Dota, 20–21.ii.2013, J.S. Noyes, NHM (Ent) 2012–91” (NHMUK014431038).

#### Diagnosis

**(male).** Head dark brown, scape yellowish brown, pedicel and flagellum brown; ventral plaque on scape ca. 0.4 × as long as scape (Fig. 27), antenna with dorsobasal setae on F1 1.5 × as long as F1; gaster with lateral tufts of pale and flattened setae on Gt_6_.

#### Description

**(male holotype MZLU:7036.1).** Length of body 1.4 mm (paratypes 1.3–1.5 mm). Head dark brown, scape yellowish brown, pedicel and flagellum brown, ventral plaque dark brown. Mesoscutum, mesoscutellum and propodeum dark brown, dorsellum pale brown. Legs with coxae and femora brown, trochanters, tibiae and tarsi yellowish brown. Gaster dark brown.

***Head*.** Length/width in frontal view 0.8; width/length in dorsal view 2.4; POL/OOL 2.4; WM/MS 1.5; MS/HE 0.5; HE/head length in frontal view 0.6; widths head/mesoscutum 1.1. ***Antenna*.** Pedicel + flagellum length/mesoscutum width 1.8; pedicel + flagellum length/head width 1.7; lengths scape/ventral plaque 2.8; ventral plaque located below the middle of scape; scape length/width 3.1; lengths scape/head (dorsal view) 0.6; scape length/HE 1.0; length/width F1, F2, F3, F4, clava: 2.6, 3.0, 3.0, 2.8, 7.6; length dorsobasal setae on F1/length F1 1.5. ***Mesosoma*.** Length/width 1.4; mesoscutum length/width 0.6; mesoscutellum length/width 0.8; widths SMG/SLG 1.1; enclosed space between SMG length/width 2.1; lengths mesoscutum/mesoscutellum 1.6; lengths mesoscutellum/dorsellum 2.9; lengths mesosoma/gaster 0.8. ***Wings*.**CC length/width 24.0; lengths CC/MV 1.1; lengths MV/ST 1.9; lengths MV/PM 3.8; lengths PM/ST 0.5; submarginal vein with seven setae (5–7 setae in paratypes) on dorsal surface. ***Gaster*.** With lateral tufts of pale and flattened setae on Gt_6_.

### Eriastichus ellipsis sp. nov.

Taxon classificationAnimaliaHymenopteraEulophidae

A4851738-2F11-58AD-8E80-9A7DBEF6E493

http://zoobank.org/B8933986-9084-433F-B5BC-3BB531D04D8E

Figure 25

#### Type locality.

Costa Rica, Heredia, Estación Biológica La Selva, 75 m, 10°26'N, 84°01'W, 24–25.ii.2011, J.S. Noyes leg.

#### Type specimen.

***Holotype*** male dried and glued to a paper card. Original labels: ”COSTA RICA, Heredia, E.B. La Selva, 75 m, 10°26'N, 84°01'W, 24–25.ii.2011, J.S. Noyes, NHM (Ent) 2011–93”, “HOLOTYPE Eriastichus ellipsis Hansson” [red printed label], (NHMUK014431039).

#### Additional type material.

***Paratype*** 1♂ with same label data as holotype (NHMUK014431040).

#### Diagnosis

**(male).** Head dark brown with part below antennal toruli yellowish brown, scape yellowish brown, pedicel and flagellum brown; ventral plaque on scape ca. 0.2 × as long as scape (Fig. 25), antenna with dorsobasal setae on F1 0.6 × as long as F1; gaster with lateral tufts of pale and flattened setae on Gt_6_.

#### Description

**(male holotype NHMUK014431039).** Length of body 1.1 mm (paratype 1.1 mm). Head dark brown with part below antennal toruli yellowish brown, scape yellowish brown, pedicel and flagellum brown, ventral plaque dark brown. Mesoscutum and mesoscutellum black with metallic tinges, dorsellum pale brown, propodeum dark brown. Legs yellowish brown. Gaster dark brown.

***Head*.** Length/width in frontal view 0.7; width/length in dorsal view 2.3; POL/OOL 3.5; WM/MS 1.3; MS/HE 0.5; HE/head length in frontal view 0.6; widths head/mesoscutum 1.2. ***Antenna*.** Pedicel + flagellum length/mesoscutum width 1.8; pedicel + flagellum length/head width 1.5; lengths scape/ventral plaque 4.8; ventral plaque located in the middle of scape; scape length/width 3.2; lengths scape/head (dorsal view) 0.5; scape length/HE 0.8; length/width F1, F2, F3, F4, clava: 1.5, 2.0, 2.2, 2.2, 6.0; length dorsobasal setae on F1/length F1 0.6. ***Mesosoma*.** Length/width 1.3; mesoscutum length/width 0.6; mesoscutellum length/width 0.7; widths SMG/SLG 1.4; enclosed space between SMG length/width 1.7; lengths mesoscutum/mesoscutellum 1.5; lengths mesoscutellum/dorsellum 2.8; lengths mesosoma/gaster 0.8. ***Wings*.**CC length/width 21.3; lengths CC/MV 1.1; lengths MV/ST 2.0; lengths MV/PM 5.0; lengths PM/ST 0.4; submarginal vein with six setae (five setae in paratype) on dorsal surface. ***Gaster*.** With lateral tufts of pale and flattened setae on Gt_6_.

### Eriastichus eminis sp. nov.

Taxon classificationAnimaliaHymenopteraEulophidae

9B6B1102-4E71-526C-85B1-AF650B9D2536

http://zoobank.org/91B5BBE0-7453-4F7D-BB97-FD1DFC02224F

Figure 31

#### Type locality.

Costa Rica, Guanacaste, Estación Pitilla, Area Conservación de Guanacaste, 10°59'N, 85°26'W, 21.ii.2011, J.S. Noyes leg.

#### Type specimen.

***Holotype*** male dried and glued to a paper card. Original labels: ”COSTA RICA, Guanacaste, Est. Pitilla, ACG, 21.ii.2011, J.S. Noyes, NHM (Ent) 2011–93”, “HOLOTYPE Eriastichus eminis Hansson” [red printed label], (NHMUK014431041).

#### Diagnosis

**(male).** Head dark brown with part below antennal toruli dark yellowish brown, scape yellowish brown, pedicel and flagellum brown; ventral plaque on scape ca. 0.2 × as long as scape (Fig. 31), antenna with dorsobasal setae on F1 1.1 × as long as F1; gaster with lateral tufts of pale and flattened setae on Gt_6_.

#### Description

**(male holotype NHMUK014431041).** Length of body 1.2 mm. Head dark brown with part below antennal toruli dark yellowish brown, scape yellowish brown, pedicel and flagellum brown, ventral plaque dark brown. Mesoscutum, mesoscutellum and propodeum dark brown, dorsellum pale brown. Legs yellowish brown. Gaster dark brown.

***Head*.** Length/width in frontal view 0.8; width/length in dorsal view 2.2; POL/OOL 2.2; WM/MS 1.5; MS/HE 0.5; HE/head length in frontal view 0.6; widths head/mesoscutum 1.2. ***Antenna*.** Pedicel + flagellum length/mesoscutum width 2.4; pedicel + flagellum length/head width 1.9; lengths scape/ventral plaque 4.5; ventral plaque located below the middle of scape; scape length/width 2.9; lengths scape/head (dorsal view) 0.5; scape length/HE 0.8; length/width F1, F2, F3, F4, clava: 3.5, 3.5, 3.3, 3.5, 8.0; length dorsobasal setae on F1/length F1 1.1. ***Mesosoma*.** Length/width 1.7; mesoscutum length/width 0.7; mesoscutellum length/width 0.9; widths SMG/SLG 1.3; enclosed space between SMG length/width 2.2; lengths mesoscutum/mesoscutellum 1.5; lengths mesoscutellum/dorsellum 2.9; lengths mesosoma/gaster 0.8. ***Wings*.**CC length/width 26.3; lengths CC/MV 1.1; lengths MV/ST 2.1; lengths MV/PM 5.7; lengths PM/ST 0.4; submarginal vein with six setae on dorsal surface. ***Gaster*.** With lateral tufts of pale and flattened setae on Gt_6_.

### Eriastichus facilis sp. nov.

Taxon classificationAnimaliaHymenopteraEulophidae

F43D02E5-73B6-54D6-A7C6-B12A8B892453

http://zoobank.org/1759534A-5BB1-4670-8391-9AD918D65A24

Figure 28

#### Type locality.

Costa Rica, Puntarenas, Estación Biológica Monteverde, 10°20'N, 84°49'W, 1540 m, 26.ii.2007, J.S. Noyes leg.

#### Type specimen.

***Holotype*** male dried and glued to a paper card. Original labels: ”Costa Rica, Puntarenas, E.B. Monteverde, 10°20'N, 84°49'W, 1540 m, 26.ii.2007, J.S. Noyes”, “HOLOTYPE Eriastichus facilis Hansson” [red printed label], (NHMUK014431042).

#### Additional type material.

***Paratypes*** (2♂♂): 1♂ “Costa Rica, Cartago, Humo, El Copal, 9°47'N, 83°45'W, 1050–1250 m, 29.ii–6.iii.2008, C. Hansson” (MZLU:7037.2); 1♂ “COSTA RICA, Alajuela, Est. Pilón, 10°43'N, 85°59'W, 700 m, 12–18.ii.2003, C. Hansson & J.A. Azofeifa” (MZLU:7037.3).

#### Diagnosis

**Figures 28–34.**
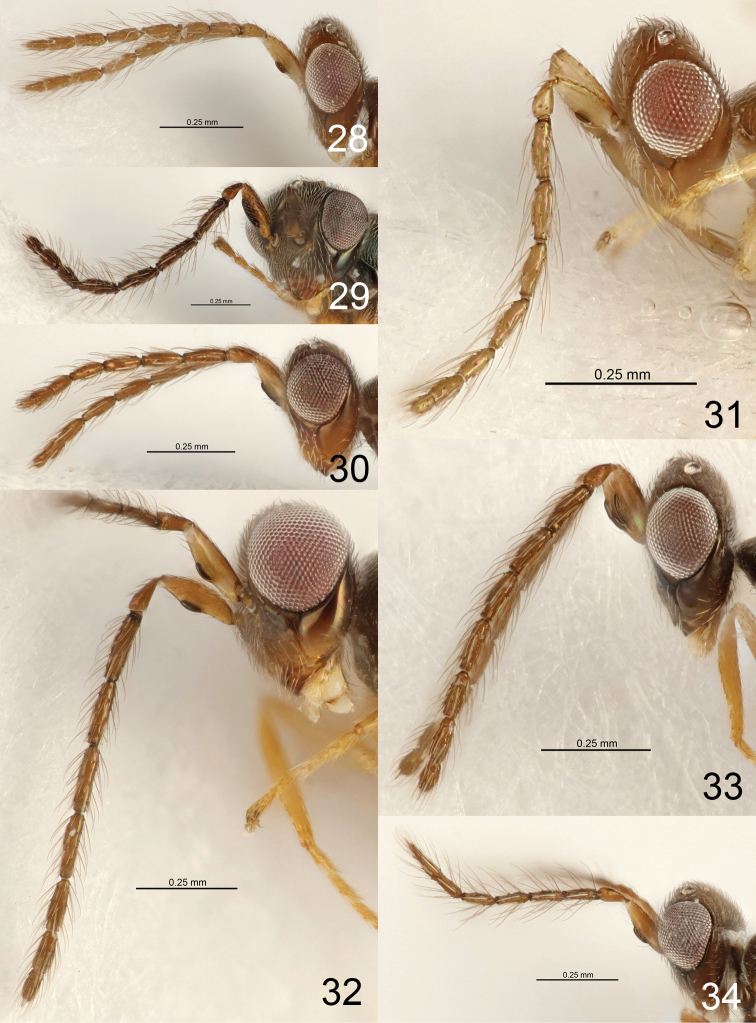
*Eriastichus* spp. head and antenna in lateral view, male holotypes **28***E. facilis***29***E. fenestris***30***E. follis***31***E. eminis***32***E. glanis***33***E. geratis***34***E. galeatis*.

**(male).** Head dark brown, scape yellowish brown, pedicel and flagellum brown; ventral plaque on scape ca. 0.2 × as long as scape (Fig. 28), antenna with dorsobasal setae on F1 0.9 × as long as F1; gaster with lateral tufts of pale and flattened setae on Gt_6_.

#### Description

**(male holotype NHMUK014431042).** Length of body 1.5 mm (paratypes 1.0–1.1 mm). Head dark brown, scape yellowish brown, pedicel and flagellum brown, ventral plaque dark brown. Mesoscutum, mesoscutellum and propodeum dark brown, dorsellum pale brown. Legs with hind coxa and hind femur dark brown, remaining parts of legs yellowish brown. Gaster dark brown.

***Head*.** Length/width in frontal view 0.8; width/length in dorsal view 2.4; POL/OOL 2.6; WM/MS 1.4; MS/HE 0.5; HE/head length in frontal view 0.6; widths head/mesoscutum 1.0. ***Antenna*.** Pedicel + flagellum length/mesoscutum width 1.7; pedicel + flagellum length/head width 1.6; lengths scape/ventral plaque 4.7; ventral plaque located below the middle of scape; scape length/width 3.5; lengths scape/head (dorsal view) 0.5; scape length/HE 0.9; length/width F1, F2, F3, F4, clava: 2.2, 2.2, 2.5, 2.5, 6.3; length dorsobasal setae on F1/length F1 0.9. ***Mesosoma*.** Length/width 1.4; mesoscutum length/width 0.6; mesoscutellum length/width 0.7; widths SMG/SLG 1.2; enclosed space between SMG length/width 2.1; lengths mesoscutum/mesoscutellum 1.4; lengths mesoscutellum/dorsellum 2.7; lengths mesosoma/gaster 0.9. ***Wings*.**CC length/width 18.0; lengths CC/MV 1.2; lengths MV/ST 2.0; lengths MV/PM 3.8; lengths PM/ST 0.5; submarginal vein with five setae on dorsal surface, in all types. ***Gaster*.** With lateral tufts of pale and flattened setae on Gt_6_.

### Eriastichus fenestris sp. nov.

Taxon classificationAnimaliaHymenopteraEulophidae

4535CE65-CEEB-5FCD-A72F-E9C2B5E18081

http://zoobank.org/68B54220-6BD6-40CE-9CC0-CEEE6962E631

Figures 5 , 29

#### Type locality.

Costa Rica, San José, San Gerardo de Dota, 9°33'N, 83°47'W, 20–21.ii.2013, J.S. Noyes leg.

#### Type specimen.

***Holotype*** male dried and glued to a paper card. Original labels: ”Costa Rica, San José, San Gerardo de Dota, 20–21.ii.2013, J.S. Noyes, NHM (Ent) 2012–91”, “HOLOTYPE Eriastichus fenestris Hansson” [red printed label], (NHMUK014431043).

#### Diagnosis

**(male).** Head black, scape brown, pedicel and flagellum dark brown; ventral plaque on scape ca. 0.6 × as long as scape (Fig. 29), antenna with dorsobasal setae on F1 0.7 × as long as F1; gaster with lateral tufts of pale and flattened setae on Gt_4-6_.

#### Description

**(male holotype NHMUK014431043).** Length of body 2.1 mm. Head black, scape brown, pedicel, flagellum and ventral plaque dark brown. Mesoscutum, mesoscutellum and dorsellum black with metallic tinges, propodeum black. Legs with fore and mid coxae yellowish brown with base dark brown, hind coxa black, remaining parts of legs yellowish brown. Gaster dark brown.

***Head*.** Length/width in frontal view 0.7; width/length in dorsal view 2.2; POL/OOL 1.8; WM/MS 1.6; MS/HE 0.6; HE/head length in frontal view 0.6; widths head/mesoscutum 1.0. ***Antenna*.** Pedicel + flagellum length/mesoscutum width 2.1; pedicel + flagellum length/head width 2.1; lengths scape/ventral plaque 1.7; ventral plaque located in the middle of scape; scape length/width 2.6; lengths scape/head (dorsal view) 0.5; scape length/HE 0.9; length/width F1, F2, F3, F4, clava: 3.2, 3.0, 2.9, 3.0, 5.8; length dorsobasal setae on F1/length F1 0.7. ***Mesosoma*.** Length/width 1.6; mesoscutum length/width 0.6; mesoscutellum length/width 0.8; widths SMG/SLG 1.2; enclosed space between SMG length/width 2.2; lengths mesoscutum/mesoscutellum 1.5; lengths mesoscutellum/dorsellum 2.3; lengths mesosoma/gaster 1.0. ***Wings*.**CC length/width 19.0; lengths CC/MV 0.9; lengths MV/ST 2.1; lengths MV/PM 5.5; lengths PM/ST 0.4; submarginal vein with ten setae on dorsal surface. ***Gaster*.** With lateral tufts of pale and flattened setae on Gt_4-6_.

### Eriastichus follis sp. nov.

Taxon classificationAnimaliaHymenopteraEulophidae

B4926812-AFF2-5C1A-AB1E-BE44E18851FB

http://zoobank.org/F9EFCF3E-87E4-4157-9AE2-ADDA7AF0E7D3

Figure 30

#### Type locality.

Costa Rica, Puntarenas, Estación Biológica Monteverde, 10°20'N, 84°49'W, 1540 m, 26.ii.2007, J.S. Noyes leg.

#### Type specimen.

***Holotype*** male dried and glued to a paper card. Original labels: ”Costa Rica, Puntarenas, E.B. Monteverde, 10°20'N, 84°49'W, 1540 m, 26.ii.2007, J.S. Noyes”, “HOLOTYPE Eriastichus follis Hansson” [red printed label], (NHMUK014431044).

#### Diagnosis

**(male).** Head dark brown with part below antennal toruli pale brown, scape and pedicel pale brown, flagellum brown; ventral plaque on scape ca. 0.6 × as long as scape (Fig. 30), antenna with dorsobasal setae on F1 0.6 × as long as F1; gaster with lateral tufts of pale and flattened setae on Gt_6_.

#### Description

**(male holotype NHMUK014431044).** Length of body 1.5 mm. Head dark brown with part below antennal toruli pale brown, scape and pedicel pale brown, flagellum brown, ventral plaque dark brown. Mesoscutum and mesoscutellum dark brown with metallic tinges, dorsellum and propodeum dark brown. Legs with hind coxa pale brown, remaining parts of legs yellowish brown. Gaster dark brown.

***Head*.** Length/width in frontal view 0.8; width/length in dorsal view 2.1; POL/OOL 1.9; WM/MS 1.2; MS/HE 0.5; HE/head length in frontal view 0.5; widths head/mesoscutum 1.2. ***Antenna*.** Pedicel + flagellum length/mesoscutum width 2.1; pedicel + flagellum length/head width 1.8; lengths scape/ventral plaque 1.7; ventral plaque located below the middle of scape; scape length/width 2.9; lengths scape/head (dorsal view) 0.5; scape length/HE 0.8; length/width F1, F2, F3, F4, clava: 2.8, 2.7, 2.7, 2.7, 6.3; length dorsobasal setae on F1/length F1 0.6. ***Mesosoma*.** Length/width 1.7; mesoscutum length/width 0.7; mesoscutellum length/width 0.8; widths SMG/SLG 1.2; enclosed space between SMG length/width 2.3; lengths mesoscutum/mesoscutellum 1.5; lengths mesoscutellum/dorsellum 2.8; lengths mesosoma/gaster 0.8. ***Wings*.**CC length/width 16.7; lengths CC/MV 0.9; lengths MV/ST 2.3; lengths MV/PM 4.9; lengths PM/ST 0.5; submarginal vein with nine setae on dorsal surface. ***Gaster*.** With lateral tufts of pale and flattened setae on Gt_6_.

### Eriastichus galeatis sp. nov.

Taxon classificationAnimaliaHymenopteraEulophidae

63079B16-A1A0-53F5-B4E7-72FBFD2F1F69

http://zoobank.org/04AE8D37-56D4-411C-88A0-04FDC46979A3

Figure 34

#### Type locality.

Costa Rica, Puntarenas, Estación Biológica Monteverde, 10°20'N, 84°49'W, 1540 m, 26.ii.2007, J.S. Noyes leg.

#### Type specimen.

***Holotype*** male dried and glued to a paper card. Original labels: ”Costa Rica, Puntarenas, E. Monteverde, 10°20'N, 84°49'W, 1540 m, 26.ii.2007, J.S. Noyes”, “HOLOTYPE Eriastichus galeatis Hansson” [red printed label], (NHMUK014431045).

#### Diagnosis

**(male).** Head dark brown, scape and pedicel pale brown, flagellum brown; ventral plaque on scape ca. 0.3 × as long as scape (Fig. 34), antenna with dorsobasal setae on F1 1.0 × as long as F1; gaster with lateral tufts of pale and flattened setae on Gt_6_.

#### Description

**(male holotype NHMUK014431045).** Length of body 1.5 mm. Head dark brown, scape and pedicel pale brown, flagellum brown, ventral plaque dark brown. Mesoscutum, mesoscutellum, dorsellum and propodeum black with metallic tinges. Legs with coxae and femora dark brown, hind tibia pale brown, remaining parts of legs yellowish brown. Gaster dark brown.

***Head*.** Length/width in frontal view 0.7; width/length in dorsal view 2.3; POL/OOL 2.1; WM/MS 1.6; MS/HE 0.5; HE/head length in frontal view 0.6; widths head/mesoscutum 1.1. ***Antenna*.** Pedicel + flagellum length/mesoscutum width 1.8; pedicel + flagellum length/head width 1.7; lengths scape/ventral plaque 3.1; ventral plaque located below the middle of scape; scape length/width 3.3; lengths scape/head (dorsal view) 0.5; scape length/HE 0.9; length/width F1, F2, F3, F4, clava: 3.0, 2.5, 2.5, 2.5, 6.3; length dorsobasal setae on F1/length F1 1.0. ***Mesosoma*.** Length/width 1.5; mesoscutum length/width 0.6; mesoscutellum length/width 0.7; widths SMG/SLG 1.3; enclosed space between SMG length/width 1.9; lengths mesoscutum/mesoscutellum 1.5; lengths mesoscutellum/dorsellum 2.7; lengths mesosoma/gaster 0.9. ***Wings*.**CC length/width 19.2; lengths CC/MV 1.0; lengths MV/ST 2.2; lengths MV/PM 3.8; lengths PM/ST 0.6; submarginal vein with eight setae on dorsal surface. ***Gaster*.** With lateral tufts of pale and flattened setae on Gt_6_.

### Eriastichus geratis sp. nov.

Taxon classificationAnimaliaHymenopteraEulophidae

21FEDC6D-03A4-596B-97E7-D51760089092

http://zoobank.org/9BF19DD1-E099-457E-AFA4-0D5D82E0BA08

Figure 33

#### Type locality.

Costa Rica, Puntarenas, San Vito, Estación Biológica Las Alturas, 1500 m, 8°57'N, 82°50'W, 17–18.ii.2012, J.S. Noyes leg.

#### Type specimen.

***Holotype*** male dried and glued to a paper card. Original labels: ”COSTA RICA, Puntarenas, San Vito, E. Las Alturas, 1500 m, 8°57'N, 82°50'W, 17–18.ii.2012, J.S. Noyes, NHM (Ent) 2012–91”, “HOLOTYPE Eriastichus geratis Hansson” [red printed label], (NHMUK014431046).

#### Additional type material.

***Paratype*** 1♂ “Costa Rica, San José, San Gerardo de Dota, 20–21.ii.2013, J.S. Noyes, NHM (Ent) 2012–91” (NHMUK014431047).

#### Diagnosis

**(male).** Head dark brown, scape and pedicel pale brown, flagellum brown; ventral plaque on scape ca. 0.4 × as long as scape (Fig. 33), antenna with dorsobasal setae on F1 0.6 × as long as F1; gaster with lateral tufts of pale and flattened setae on Gt_6_.

#### Description

**(male holotype NHMUK014431046).** Length of body 1.8 mm (paratype 1.7 mm). Head dark brown, scape and pedicel pale brown, flagellum brown, ventral plaque dark brown. Mesoscutum and mesoscutellum black with metallic tinges, dorsellum dark brown, propodeum black. Legs with coxae, mid and hind femora dark brown, fore femur pale brown, remaining parts of legs yellowish brown. Gaster dark brown.

***Head*.** Length/width in frontal view 0.8; width/length in dorsal view 2.4; POL/OOL 1.9; WM/MS 1.6; MS/HE 0.6; HE/head length in frontal view 0.5; widths head/mesoscutum 1.0. ***Antenna*.** Pedicel + flagellum length/mesoscutum width 1.8; pedicel + flagellum length/head width 1.8; lengths scape/ventral plaque 2.5; ventral plaque located in the middle of scape; scape length/width 2.8; lengths scape/head (dorsal view) 0.5; scape length/HE 0.9; length/width F1, F2, F3, F4, clava: 3.2, 2.8, 3.2, 3.0, 7.7; length dorsobasal setae on F1/length F1 0.6. ***Mesosoma*.** Length/width 1.5; mesoscutum length/width 0.6; mesoscutellum length/width 0.7; widths SMG/SLG 1.2; enclosed space between SMG length/width 1.9; lengths mesoscutum/mesoscutellum 1.5; lengths mesoscutellum/dorsellum 2.5; lengths mesosoma/gaster 0.8. ***Wings*.**CC length/width 19.3; lengths CC/MV 1.1; lengths MV/ST 2.2; lengths MV/PM 4.3; lengths PM/ST 0.5; submarginal vein with six setae on dorsal surface, in both types. ***Gaster*.** With lateral tufts of pale and flattened setae on Gt_6_.

### Eriastichus glanis sp. nov.

Taxon classificationAnimaliaHymenopteraEulophidae

575439FE-F81A-5CB2-84D1-267A246FE884

http://zoobank.org/CC8A526B-4C9D-4B48-8503-9CE11D084C54

Figure 32

#### Type locality.

Costa Rica, Limón, Reserva Biológica Hitoy-Cerere, Headquarters, 100 m, 9°40'N, 83°02'W, 24–26.ii.2008, J.S. Noyes leg.

#### Type specimen.

***Holotype*** male dried and glued to a paper card. Original labels: ”Costa Rica, Limón, R.B. Hitoy-Cerere, HQ, 100 m, 9°40'N, 83°02'W, 24–26.ii.2008, J.S. Noyes, BMNH(Ent) 2010–21”, “HOLOTYPE Eriastichus glanis Hansson” [red printed label], (NHMUK014431048).

#### Diagnosis

**(male).** Head dark brown, scape yellowish brown, pedicel pale brown, flagellum brown; ventral plaque on scape ca. 0.3 × as long as scape (Fig. 32), antenna with dorsobasal setae on F1 0.5 × as long as F1; gaster with lateral tufts of pale and flattened setae on Gt_6_.

#### Description

**(male holotype NHMUK014431048).** Length of body 2.0 mm. Head dark brown, scape yellowish brown, pedicel pale brown, flagellum brown, ventral plaque dark brown. Mesoscutum and mesoscutellum black with metallic tinges, dorsellum and propodeum dark brown. Legs yellowish brown, hind coxa with base dark brown. Gaster dark brown.

***Head*.** Length/width in frontal view 0.7; width/length in dorsal view 2.1; POL/OOL 1.3; WM/MS 1.8; MS/HE 0.4; HE/head length in frontal view 0.6; widths head/mesoscutum 1.0. ***Antenna*.** Pedicel + flagellum length/mesoscutum width 2.4; pedicel + flagellum length/head width 2.0; lengths scape/ventral plaque 4.0; ventral plaque located in the middle of scape; scape length/width 3.1; lengths scape/head (dorsal view) 0.5; scape length/HE 0.8; length/width F1, F2, F3, F4, clava: 4.0, 4.0, 4.2, 3.8, 8.2; length dorsobasal setae on F1/length F1 0.5. ***Mesosoma*.** Length/width 1.6; mesoscutum length/width 0.7; mesoscutellum length/width 0.9; widths SMG/SLG 1.7; enclosed space between SMG length/width 2.2; lengths mesoscutum/mesoscutellum 1.4; lengths mesoscutellum/dorsellum 3.3; lengths mesosoma/gaster 0.7. ***Wings*.**CC length/width 18.7; lengths CC/MV 0.8; lengths MV/ST 2.9; lengths MV/PM 4.7; lengths PM/ST 0.6; submarginal vein with nine setae on dorsal surface. ***Gaster*.** With lateral tufts of pale and flattened setae on Gt_6_.

### Eriastichus hilaris sp. nov.

Taxon classificationAnimaliaHymenopteraEulophidae

ED2907DD-7E4B-5E46-B731-C5DDBFB6623F

http://zoobank.org/9FFEC823-0436-45EE-BA8F-54FD1FB5E740

Figure 35

#### Type locality.

Costa Rica, San José, San Gerardo de Dota, 9°33'N, 83°47'W, 20–21.ii.2013, J.S. Noyes leg.

#### Type specimen.

***Holotype*** male dried and glued to a paper card. Original labels: ”Costa Rica, San José, San Gerardo de Dota, 20–21.ii.2013, J.S. Noyes, NHM (Ent) 2012–91”, “HOLOTYPE Eriastichus hilaris Hansson” [red printed label], (NHMUK014431049).

#### Diagnosis

**Figures 35–40.**
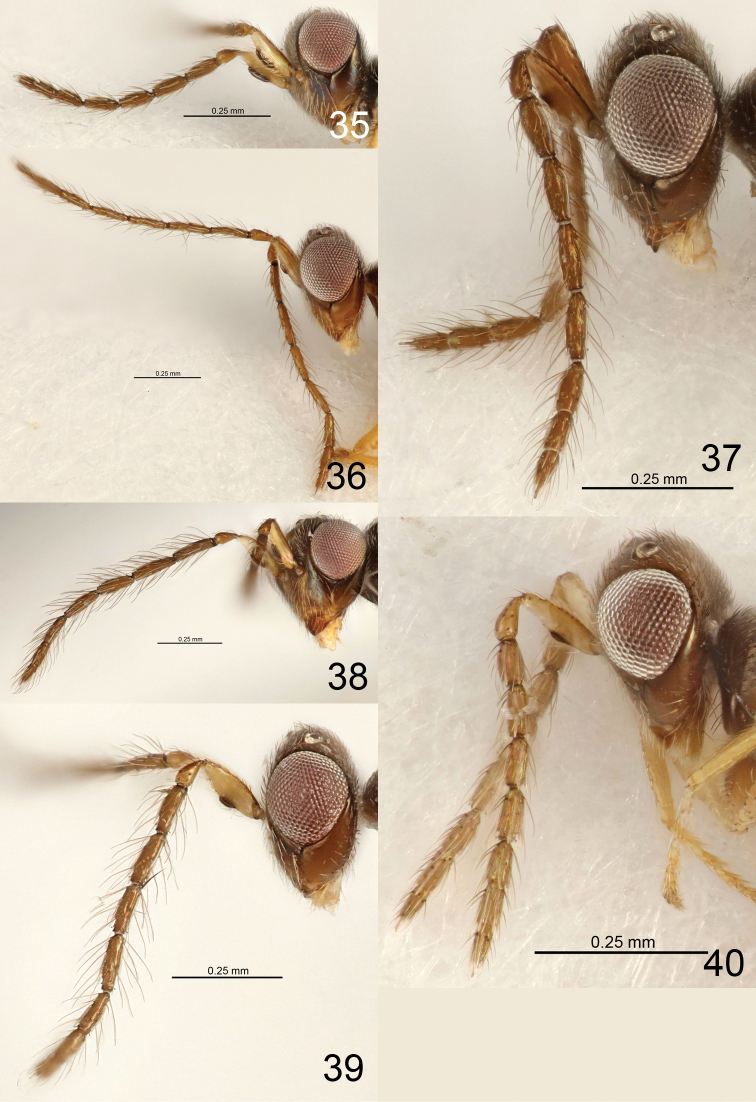
*Eriastichus* spp. head and antenna in lateral view, male holotypes **35***E. hilaris***36***E. maniatis***37***E. johnlasallei***38***E. nebulis***39***E. neonis***40***E. johnnoyesi*.

**(male).** Head black with part below antennal toruli pale brown, scape yellowish brown, pedicel pale brown, flagellum dark brown; ventral plaque on scape ca. 0.5 × as long as scape (Fig. 35), antenna with dorsobasal setae on F1 0.4 × as long as F1; gaster with lateral tufts of pale and flattened setae on Gt_5-6_.

#### Description

**(male holotype NHMUK014431049).** Length of body 1.7 mm. Head black with part below antennal toruli pale brown, scape yellowish brown, pedicel pale brown, flagellum dark brown, ventral plaque dark brown. Mesoscutum and mesoscutellum black with metallic tinges, dorsellum dark brown, propodeum black. Legs with hind coxa with basal ½ dark brown and apical ½ yellowish brown, remaining parts of legs yellowish brown. Gaster dark brown.

***Head*.** Length/width in frontal view 0.8; width/length in dorsal view 2.0; POL/OOL 2.0; WM/MS 1.4; MS/HE 0.6; HE/head length in frontal view 0.5; widths head/mesoscutum 1.2. ***Antenna*.** Pedicel + flagellum length/mesoscutum width 1.9; pedicel + flagellum length/head width 1.7; lengths scape/ventral plaque 2.2; ventral plaque located above the middle of scape; scape length/width 2.9; lengths scape/head (dorsal view) 0.5; scape length/HE 0.9; length/width F1, F2, F3, F4, clava: 2.3, 2.4, 2.1, 2.2, 4.9; length dorsobasal setae on F1/length F1 0.4. ***Mesosoma*.** Length/width 1.6; mesoscutum length/width 0.5; mesoscutellum length/width 0.8; widths SMG/SLG 1.0; enclosed space between SMG length/width 2.4; lengths mesoscutum/mesoscutellum 1.2; lengths mesoscutellum/dorsellum 2.9; lengths mesosoma/gaster 0.8. ***Wings*.**CC length/width 17.3; lengths CC/MV 1.0; lengths MV/ST 2.2; lengths MV/PM 4.5; lengths PM/ST 0.5; submarginal vein with nine setae on dorsal surface. ***Gaster***. With lateral tufts of pale and flattened setae on Gt_5-6_.

### Eriastichus johnlasallei sp. nov.

Taxon classificationAnimaliaHymenopteraEulophidae

D7A1D624-6B25-5743-813E-0F0030FD4737

http://zoobank.org/2A25525C-B5A5-471F-9697-A6936C8FB55E

Figure 37

#### Type locality.

Costa Rica, Cartago, Humo, El Copal, 9°47'N, 83°45'W, 1050–1250 m, 29.ii–6.iii.2008, C. Hansson leg.

#### Type specimen.

***Holotype*** male dried and glued to a paper card. Original labels: ”Costa Rica, Cartago, Humo, El Copal, 9°47'N, 83°45'W, 1050–1250 m, 29.ii–6.iii.2008, C. Hansson”, “HOLOTYPE Eriastichus johnlasallei Hansson” [red printed label], (MZLU:7038.1).

#### Diagnosis

**(male).** Head and antenna dark brown; ventral plaque on scape ca. 0.2 × as long as scape (Fig. 37), antenna with dorsobasal setae on F1 0.8 × as long as F1; gaster with lateral tufts of pale and flattened setae on Gt_6_.

#### Description

**(male holotype MZLU:7038.1).** Length of body 1.5 mm. Head and antenna dark brown, ventral plaque black. Mesoscutum, mesoscutellum and propodeum dark brown; dorsellum pale brown. Legs yellowish brown, coxae and hind femur brown. Gaster dark brown.

***Head*.** Length/width in frontal view 0.7; width/length in dorsal view 2.3; POL/OOL 2.0; WM/MS 1.7; MS/HE 0.5; HE/head length in frontal view 0.6; widths head/mesoscutum 1.2. ***Antenna*.** Pedicel + flagellum length/mesoscutum width 2.0; pedicel + flagellum length/head width 1.7; lengths scape/ventral plaque 4.7; ventral plaque located in the middle of scape; scape length/width 3.5; lengths scape/head (dorsal view) 0.6; scape length/HE 0.9; length/width F1, F2, F3, F4, clava: 2.5, 2.7, 2.8, 2.8, 6.2; length dorsobasal setae on F1/length F1 0.8. ***Mesosoma*.** Length/width 1.5; mesoscutum length/width 0.5; mesoscutellum length/width 0.8; widths SMG/SLG 1.6; enclosed space between SMG length/width 1.9; lengths mesoscutum/mesoscutellum 1.2; lengths mesoscutellum/dorsellum 3.6; lengths mesosoma/gaster 0.9. ***Wings*.**CC length/width 24.0; lengths CC/MV 0.9; lengths MV/ST 2.2; lengths MV/PM 4.7; lengths PM/ST 0.5; submarginal vein with seven setae on dorsal surface. ***Gaster*.** With lateral tufts of pale and flattened setae on Gt_6_.

#### Etymology.

Named after John La Salle, fellow eulophid taxonomist who described *Eriastichus*.

### Eriastichus johnnoyesi sp. nov.

Taxon classificationAnimaliaHymenopteraEulophidae

48DA0DF7-AFB0-5BE6-B0BA-7446C0F4ED49

http://zoobank.org/1F78E014-A16C-4D6F-8CFB-FC8EEF163665

Figure 40

#### Type locality.

Costa Rica, Heredia, Estación Biológica La Selva, 75 m, 10°26'N, 84°01'W, 24–25.ii.2011, J.S. Noyes leg.

#### Type specimen.

***Holotype*** male dried and glued to a paper card. Original labels: ”COSTA RICA, Heredia, E. La Selva, 75 m, 10°26'N, 84°01'W, 24–25.ii.2011, J.S. Noyes, NHM (Ent) 2011–93”, “HOLOTYPE Eriastichus johnnoyesi Hansson” [red printed label], (NHMUK014431050.

#### Additional type material.

***Paratypes*** 2♂♂ from same locality as holotype but collected 30–31.iii.2002 (1♂, MZLU:7039.2), 23–24.ii.2005 (1♂, NHMUK014431051).

#### Diagnosis

**(male).** Head dark brown, scape yellowish brown, pedicel and flagellum brown; ventral plaque on scape ca. 0.2 × as long as scape (Fig. 40), antenna with dorsobasal setae on F1 0.6 × as long as F1; gaster with lateral tufts of pale and flattened setae on Gt_6_.

#### Description

**(male holotype NHMUK014431050).** Length of body 1.1 mm (paratypes 1.0 mm). Head dark brown, scape yellowish brown, pedicel and flagellum brown, ventral plaque dark brown. Mesoscutum and mesoscutellum black, dorsellum and propodeum dark brown. Legs with coxae and hind femur dark brown, remaining parts of legs yellowish brown. Gaster dark brown.

***Head*.** Length/width in frontal view 0.7; width/length in dorsal view 2.2; POL/OOL 3.0; WM/MS 1.3; MS/HE 0.5; HE/head length in frontal view 0.6; widths head/mesoscutum 1.2. ***Antenna*.** Pedicel + flagellum length/mesoscutum width 1.8; pedicel + flagellum length/head width 1.6; lengths scape/ventral plaque 5.0; ventral plaque located below the middle of scape; scape length/width 3.3; lengths scape/head (dorsal view) 0.5; scape length/HE 0.9; length/width F1, F2, F3, F4, clava: 2.3, 2.5, 2.3, 3.0, 7.8; length dorsobasal setae on F1/length F1 0.6. ***Mesosoma*.** Length/width 1.4; mesoscutum length/width 0.6; mesoscutellum length/width 0.7; widths SMG/SLG 1.4; enclosed space between SMG length/width 1.6; lengths mesoscutum/mesoscutellum 1.6; lengths mesoscutellum/dorsellum 2.6; lengths mesosoma/gaster 0.8. ***Wings*.**CC length/width 18.8; lengths CC/MV 0.9; lengths MV/ST 2.0; lengths MV/PM 4.0; lengths PM/ST 0.5; submarginal vein with four setae (four setae in paratypes) on dorsal surface. ***Gaster*.** With lateral tufts of pale and flattened setae on Gt_6_.

#### Etymology.

Named after John S. Noyes (NHMUK), collector of the material of this and several other species described here.

### Eriastichus maniatis sp. nov.

Taxon classificationAnimaliaHymenopteraEulophidae

5FE053F0-5C76-50CA-AA18-3F8DC7402349

http://zoobank.org/B3BA25D5-CA2F-4B3D-AB17-B8AAF4848F7D

Figure 36

#### Type locality.

Costa Rica, Puntarenas, San Vito, Las Cruces, 8°46'N, 82°57'W, 1300 m, 15–16.ii.2006, J.S. Noyes leg.

#### Type specimen.

***Holotype*** male dried and glued to a paper card. Original labels: ”COSTA RICA, Puntarenas, San Vito, Las Cruces, 8°46'N, 82°57'W, 1300 m, 15–16.ii.2006, J.S. Noyes”, “HOLOTYPE Eriastichus maniatis Hansson” [red printed label], (NHMUK014431052).

#### Diagnosis

**(male).** Head dark brown with parts above mouth yellowish brown, scape yellowish brown, pedicel and flagellum brown; ventral plaque on scape ca. 0.3 × as long as scape (Fig. 36), antenna with dorsobasal setae on F1 0.6 × as long as F1; gaster with lateral tufts of pale and flattened setae on Gt_6_.

#### Description

**(male holotype NHMUK014431052).** Length of body 1.5 mm. Head dark brown with parts above mouth yellowish brown, scape yellowish brown, pedicel and flagellum brown, ventral plaque dark brown. Mesoscutum and mesoscutellum black with metallic tinges, dorsellum and propodeum dark brown. Legs yellowish brown. Gaster dark brown.

***Head*.** Length/width in frontal view 0.8; width/length in dorsal view 2.2; POL/OOL 2.1; WM/MS 1.4; MS/HE 0.5; HE/head length in frontal view 0.6; widths head/mesoscutum 1.3. ***Antenna*.** Pedicel + flagellum length/mesoscutum width 2.6; pedicel + flagellum length/head width 2.1; lengths scape/ventral plaque 3.8; ventral plaque located below the middle of scape; scape length/width 3.8; lengths scape/head (dorsal view) 0.5; scape length/HE 0.9; length/width F1, F2, F3, F4, clava: 3.7, 3.8, 3.8, 3.7, 8.3; length dorsobasal setae on F1/length F1 0.6. ***Mesosoma*.** Length/width 1.6; mesoscutum length/width 0.5; mesoscutellum length/width 0.8; widths SMG/SLG 1.0; enclosed space between SMG length/width 2.3; lengths mesoscutum/mesoscutellum 1.1; lengths mesoscutellum/dorsellum 3.1; lengths mesosoma/gaster 0.9. ***Wings*.**CC length/width 21.7; lengths CC/MV 0.9; lengths MV/ST 2.5; lengths MV/PM 3.4; lengths PM/ST 0.7; submarginal vein with seven setae on dorsal surface. ***Gaster*.** With lateral tufts of pale and flattened setae on Gt_6_.

### Eriastichus masneri

Taxon classificationAnimaliaHymenopteraEulophidae

La Salle

F74ACE8A-43DC-5EFA-903F-36E197F4F067

Eriastichus masneri La Salle, 1994: 207. Holotype female in CNC, not examined.

#### Diagnosis.

Mesoscutellum with parts lateral to submedian grooves strongly setose (as in Fig. 6); median propodeum hairy (fig. 30 in La Salle (1994)).

#### Description.

See La Salle (1994).

#### Distribution.

Dominican Republic (La Salle 1994).

### Eriastichus nakos

Taxon classificationAnimaliaHymenopteraEulophidae

La Salle

D96CAEA9-C99A-50F4-AC30-8157D288F78D

Eriastichus nakos La Salle, 1994: 208. Holotype female in CNC, not examined.

#### Diagnosis.

Mesoscutellum with parts lateral to submedian grooves strongly setose (as in Fig. 6); median propodeum bare (as in Fig. 5).

#### Description.

See La Salle (1994).

#### Distribution.

Dominican Republic, Ecuador (La Salle 1994).

### Eriastichus nebulis sp. nov.

Taxon classificationAnimaliaHymenopteraEulophidae

63D6C534-766B-5333-A3D7-C6183BB11496

http://zoobank.org/5759023B-4DDD-48BC-BC00-CF7B4ACC38F4

Figure 38

#### Type locality.

Costa Rica, Puntarenas, San Vito, Estación Biológica Las Alturas, 1500 m, 8°57'N, 82°50'W, 17–18.ii.2012, J.S. Noyes leg.

#### Type specimen.

***Holotype*** male dried and glued to a paper card. Original labels: ”COSTA RICA, Puntarenas, San Vito, E. Las Alturas, 1500 m, 8°57'N, 82°50'W, 17–18.ii.2012, J.S. Noyes, NHM (Ent) 2012–91”, “HOLOTYPE Eriastichus nebulis Hansson” [red printed label], (NHMUK014431053).

#### Diagnosis

**(male).** Head dark brown with metallic tinges, scape yellowish brown, pedicel and flagellum brown; ventral plaque on scape ca. 0.3 × as long as scape (Fig. 38), antenna with dorsobasal setae on F1 0.6 × as long as F1; gaster with lateral tufts of pale and flattened setae on Gt_6_.

#### Description

**(male holotype NHMUK014431053).** Length of body 1.9 mm. Head dark brown with metallic tinges, scape yellowish brown, pedicel and flagellum brown, ventral plaque dark brown. Mesoscutum and mesoscutellum black with metallic tinges, dorsellum and propodeum dark brown. Legs yellowish brown. Gaster dark brown.

***Head*.** Length/width in frontal view 0.7; width/length in dorsal view 2.2; POL/OOL 2.3; WM/MS 1.6; MS/HE 0.5; HE/head length in frontal view 0.6; widths head/mesoscutum 1.2. ***Antenna*.** Pedicel + flagellum length/mesoscutum width 2.3; pedicel + flagellum length/head width 1.9; lengths scape/ventral plaque 3.0; ventral plaque located below the middle of scape; scape length/width 3.0; lengths scape/head (dorsal view) 0.5; scape length/HE 0.9; length/width F1, F2, F3, F4, clava: 3.6, 3.8, 3.3, 3.3, 7.8; length dorsobasal setae on F1/length F1 0.6. ***Mesosoma*.** Length/width 1.7; mesoscutum length/width 0.7; mesoscutellum length/width 0.8; widths SMG/SLG 1.2; enclosed space between SMG length/width 2.2; lengths mesoscutum/mesoscutellum 1.5; lengths mesoscutellum/dorsellum 2.9; lengths mesosoma/gaster 0.7. ***Wings*.**CC length/width 20.0; lengths CC/MV 1.0; lengths MV/ST 2.0; lengths MV/PM 3.8; lengths PM/ST 0.5; submarginal vein with eight setae on dorsal surface. ***Gaster*.** With lateral tufts of pale and flattened setae on Gt_6_.

### Eriastichus neonis sp. nov.

Taxon classificationAnimaliaHymenopteraEulophidae

1F90125A-89D6-5562-8EA3-7294B2A37AE4

http://zoobank.org/2D31B245-9233-4289-AD36-D0111F016CC2

Figure 39

#### Type locality.

Costa Rica, Cartago, Humo, El Copal, 9°47'N, 83°45'W, 1050–1250 m, 29.ii–6.iii.2008, C. Hansson leg.

#### Type specimen.

***Holotype*** male dried and glued to a paper card. Original labels: ”Costa Rica, Cartago, Humo, El Copal, 9°47'N, 83°45'W, 1050–1250 m, 29.ii–6.iii.2008, C. Hansson”, “HOLOTYPE Eriastichus neonis Hansson” [red printed label], (MZLU:7040.1).

#### Additional type material.

***Paratypes*** (3♂♂): 1♂ with same label data as holotype (MZLU:7040.2); 1♂ “COSTA RICA, Heredia, E. La Selva, 75 m, LN 264463/532850, 30–31.iii.2002, swept, J. Azofeifa” (MZUCR:01694); 1♂ “COSTA RICA, Puntarenas, San Vito, E. Las Alturas, 1500 m, 8°57'N, 82°50'W, 17–18.ii.2012, J.S. Noyes, NHM (Ent) 2012–91” (NHMUK014431054).

#### Diagnosis

**(male).** Head dark brown, scape yellowish brown, pedicel and flagellum brown; ventral plaque on scape ca. 0.3 × as long as scape (Fig. 39), antenna with dorsobasal setae on F1 0.7 × as long as F1; gaster with lateral tufts of pale and flattened setae on Gt_6_.

#### Description

**(male holotype MZLU:7040.1).** Length of body 1.5 mm (paratypes 1.3–1.6 mm). Head dark brown, scape yellowish brown, pedicel and flagellum brown, ventral plaque dark brown. Mesoscutum, mesoscutellum and propodeum black, dorsellum dark brown. Legs yellowish brown. Gaster dark brown.

***Head*.** Length/width in frontal view 0.8; width/length in dorsal view 1.9; POL/OOL 2.5; WM/MS 1.6; MS/HE 0.5; HE/head length in frontal view 0.6; widths head/mesoscutum 1.2. ***Antenna*.** Pedicel + flagellum length/mesoscutum width 2.5; pedicel + flagellum length/head width 2.1; lengths scape/ventral plaque 4.0; ventral plaque located below the middle of scape; scape length/width 3.0; lengths scape/head (dorsal view) 0.5; scape length/HE 0.8; length/width F1, F2, F3, F4, clava: 3.0, 3.0, 3.0, 3.0, 7.0; length dorsobasal setae on F1/length F1 0.7. ***Mesosoma*.** Length/width 1.8; mesoscutum length/width 0.7; mesoscutellum length/width 0.9; widths SMG/SLG 1.2; enclosed space between SMG length/width 2.1; lengths mesoscutum/mesoscutellum 1.5; lengths mesoscutellum/dorsellum 2.6; lengths mesosoma/gaster 0.8. ***Wings*.**CC length/width 23.0; lengths CC/MV 0.9; lengths MV/ST 2.1; lengths MV/PM 5.0; lengths PM/ST 0.4; submarginal vein with six setae on dorsal surface (6–7 setae in paratypes). ***Gaster*.** With lateral tufts of pale and flattened setae on Gt_6_.

### Eriastichus nexilis sp. nov.

Taxon classificationAnimaliaHymenopteraEulophidae

7615F4A9-8B5F-5826-A1C9-B6D84FA66CAA

http://zoobank.org/A8B195EF-FC82-4D9C-86DF-DA156C09D23F

Figure 41

#### Type locality.

Costa Rica, Heredia, Estación Biológica La Selva, 75 m, 10°26'N, 84°01'W, 28–29.ii.2008, J.S. Noyes leg.

#### Type specimen.

***Holotype*** male dried and glued to a paper card. Original labels: ”COSTA RICA, Heredia, E. La Selva, 75 m, 10°26'N, 84°01'W, 28–29.ii.2008, J.S. Noyes, NHM (Ent) 2010–21”, “HOLOTYPE Eriastichus nexilis Hansson” [red printed label], (NHMUK014431055).

#### Additional type material.

***Paratype*** 1♂ “Costa Rica, Cartago, Humo, El Copal, 9°47'N, 83°45'W, 1050–1250 m, 29.ii–6.iii.2008, C. Hansson” (MZLU:7041.2).

#### Diagnosis

**Figures 41–50.**
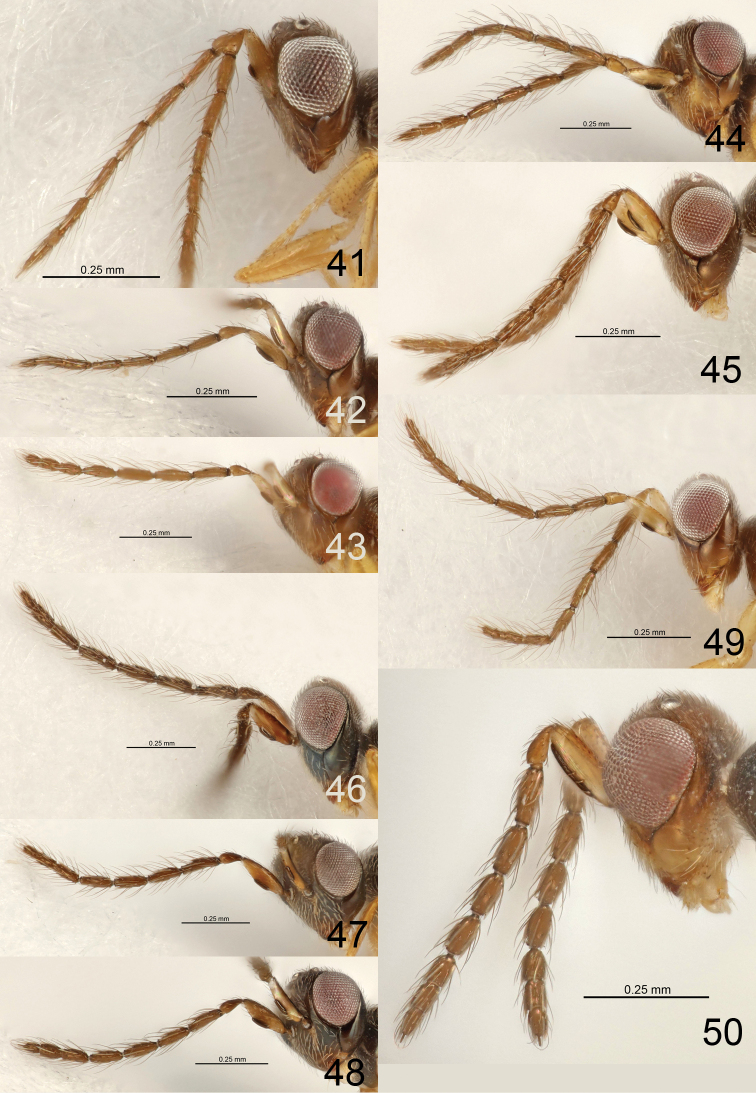
*Eriastichus* spp. head and antenna in lateral view, male holotypes **41***E. nexilis***42***E. oasis***43***E. ononis***44***E. novalis***45***E. nugalis***46***E. parabilis***47***E. rivalis***48***E. sodalis***49***E. orestis***50***E. pallidops*.

**(male).** Head dark brown, scape pale brown, pedicel and flagellum brown; ventral plaque on scape ca. 0.2 × as long as scape (Fig. 41), antenna with dorsobasal setae on F1 0.9 × as long as F1; gaster with lateral tufts of pale and flattened setae on Gt_6_.

#### Description

**(male holotype NHMUK014431055).** Length of body 1.1 mm (paratype 1.2 mm). Head dark brown, scape pale brown, pedicel and flagellum brown, ventral plaque dark brown. Mesoscutum and mesoscutellum black with metallic tinges, dorsellum dark brown, propodeum black. Legs yellowish brown. Gaster dark brown.

***Head*.** Length/width in frontal view 0.8; width/length in dorsal view 2.2; POL/OOL 2.0; WM/MS 1.6; MS/HE 0.5; HE/head length in frontal view 0.6; widths head/mesoscutum 1.1. ***Antenna*.** Pedicel + flagellum length/mesoscutum width 2.3; pedicel + flagellum length/head width 1.9; lengths scape/ventral plaque 4.2; ventral plaque located below the middle of scape; scape length/width 3.0; lengths scape/head (dorsal view) 0.5; scape length/HE 0.9; length/width F1, F2, F3, F4, clava: 3.3, 3.8, 3.5, 3.5, 8.5; length dorsobasal setae on F1/length F1 0.9. ***Mesosoma*.** Length/width 1.4; mesoscutum length/width 0.6; mesoscutellum length/width 0.8; widths SMG/SLG 1.1; enclosed space between SMG length/width 2.1; lengths mesoscutum/mesoscutellum 1.4; lengths mesoscutellum/dorsellum 3.2; lengths mesosoma/gaster 0.7. ***Wings*.**CC length/width 22.5; lengths CC/MV 1.1; lengths MV/ST 1.9; lengths MV/PM 4.0; lengths PM/ST 0.5; submarginal vein with six setae on dorsal surface (five setae in paratype). ***Gaster*.** With lateral tufts of pale and flattened setae on Gt_6_.

### Eriastichus novalis sp. nov.

Taxon classificationAnimaliaHymenopteraEulophidae

4981AD43-5E7D-546A-BA16-0C6400CD62BD

http://zoobank.org/A656E7CC-7D18-4A45-BDB8-F5F05D7C539D

Figure 44

#### Type locality.

Costa Rica, San José, San Gerardo de Dota, 9°33'N, 83°47'W, 20–21.ii.2013, J.S. Noyes leg.

#### Type specimen.

***Holotype*** male dried and glued to a paper card. Original labels: ”Costa Rica, San José, San Gerardo de Dota, 20–21.ii.2013, J.S. Noyes, NHM (Ent) 2012–91”, “HOLOTYPE Eriastichus novalis Hansson” [red printed label], (NHMUK014431056).

#### Diagnosis

**(male).** Head dark brown with part below antennal toruli yellowish brown, scape yellowish brown, pedicel pale brown, flagellum brown; ventral plaque on scape ca. 0.4 × as long as scape (Fig. 44), antenna with dorsobasal setae on F1 0.9 × as long as F1; gaster with lateral tufts of pale and flattened setae on Gt_6_.

#### Description

**(male holotype NHMUK014431056).** Length of body 1.7 mm. Head dark brown with part below antennal toruli yellowish brown, scape yellowish brown, pedicel pale brown, flagellum brown, ventral plaque dark brown. Mesoscutum and mesoscutellum dark brown with metallic tinges, dorsellum pale brown, propodeum dark brown. Legs yellowish brown. Gaster dark brown.

***Head*.** Length/width in frontal view 0.7; width/length in dorsal view 2.3; POL/OOL 1.8; WM/MS 1.6; MS/HE 0.5; HE/head length in frontal view 0.6; widths head/mesoscutum 1.0. ***Antenna*.** Pedicel + flagellum length/mesoscutum width 2.0; pedicel + flagellum length/head width 1.8; lengths scape/ventral plaque 2.5; ventral plaque located below the middle of scape; scape length/width 2.5; lengths scape/head (dorsal view) 0.5; scape length/HE 0.8; length/width F1, F2, F3, F4, clava: 3.2, 3.0, 2.7, 2.8, 6.5; length dorsobasal setae on F1/length F1 0.9. ***Mesosoma*.** Length/width 1.4; mesoscutum length/width 0.6; mesoscutellum length/width 1.9; widths SMG/SLG 1.0; enclosed space between SMG length/width 2.0; lengths mesoscutum/mesoscutellum 1.6; lengths mesoscutellum/dorsellum 2.6; lengths mesosoma/gaster 0.7. ***Wings*.**CC length/width 23.3; lengths CC/MV 1.0; lengths MV/ST 2.1; lengths MV/PM 5.4; lengths PM/ST 0.4; submarginal vein with ten setae on dorsal surface. ***Gaster*.** With lateral tufts of pale and flattened setae on Gt_6_.

### Eriastichus nugalis sp. nov.

Taxon classificationAnimaliaHymenopteraEulophidae

EE56002B-F779-52F5-958E-8E7CF130D489

http://zoobank.org/07A69A24-BEB7-4C6C-BA98-953B99362A62

Figure 45

#### Type locality.

Costa Rica, Cartago, Humo, El Copal, 9°47'N, 83°45'W, 1050–1250 m, 29.ii–6.iii.2008, C. Hansson leg.

#### Type specimen.

***Holotype*** male dried and glued to a paper card. Original labels: ”Costa Rica, Cartago, Humo, El Copal, 9°47'N, 83°45'W, 1050–1250 m, 29.ii–6.iii.2008, C. Hansson”, “HOLOTYPE Eriastichus nugalis Hansson” [red printed label], (MZLU:7042.1).

#### Additional type material.

***Paratypes*** (2♂♂): 1♂ with same label data as holotype (MZLU:7042.2); 1♂ “COSTA RICA, Puntarenas, San Vito, E.B. Las Alturas, 1500 m, 8°57'N, 82°50'W, 17–18.ii.2012, J.S. Noyes, NHM (Ent) 2012–91” (NHMUK014431057).

#### Diagnosis

**(male).** Head dark brown, scape yellowish brown, pedicel pale brown, flagellum dark brown; ventral plaque on scape ca. 0.4 × as long as scape (Fig. 45), antenna with dorsobasal setae on F1 0.5 × as long as F1; gaster with lateral tufts of pale and flattened setae on Gt_6_.

#### Description

**(male holotype MZLU:7042.1).** Length of body 1.8 mm (paratypes 1.5–1.6 mm). Head dark brown, scape yellowish brown, pedicel pale brown, flagellum dark brown, ventral plaque dark brown. Mesoscutum, mesoscutellum and propodeum dark brown, dorsellum pale brown. Legs with fore and mid coxae yellowish brown with base brown, hind coxa, remaining parts of legs yellowish brown. Gaster dark brown.

***Head*.** Length/width in frontal view 0.8; width/length in dorsal view 2.1; POL/OOL 1.8; WM/MS 1.9; MS/HE 0.5; HE/head length in frontal view 0.6; widths head/mesoscutum 1.1. ***Antenna*.** Pedicel + flagellum length/mesoscutum width 2.1; pedicel + flagellum length/head width 1.9; lengths scape/ventral plaque 2.5; ventral plaque located in the middle of scape; scape length/width 3.0; lengths scape/head (dorsal view) 0.6; scape length/HE 0.9; length/width F1, F2, F3, F4, clava: 2.5, 2.5, 2.5, 2.5, 5.5; length dorsobasal setae on F1/length F1 0.5. ***Mesosoma*.** Length/width 1.6; mesoscutum length/width 0.6; mesoscutellum length/width 0.8; widths SMG/SLG 1.0; enclosed space between SMG length/width 2.3; lengths mesoscutum/mesoscutellum 1.4; lengths mesoscutellum/dorsellum 2.8; lengths mesosoma/gaster 0.7. ***Wings*.**CC length/width 30.0; lengths CC/MV 1.1; lengths MV/ST 1.9; lengths MV/PM 4.9; lengths PM/ST 0.4; submarginal vein with eight setae on dorsal surface (paratypes with seven setae). ***Gaster*.** With lateral tufts of pale and flattened setae on Gt_6_.

### Eriastichus oasis sp. nov.

Taxon classificationAnimaliaHymenopteraEulophidae

8F0BCA5D-4626-545D-BB27-9AAC071B397C

http://zoobank.org/3008B625-977E-4985-90B1-81A4783D1BB3

Figure 42

#### Type locality.

Costa Rica, Heredia, Estación Biológica La Selva, 75 m, 10°26'N, 84°01'W, 22–24.ii.2012, J.S. Noyes leg.

#### Type specimen.

***Holotype*** male dried and glued to a paper card. Original labels: ”COSTA RICA, Heredia, E.B. La Selva, 75 m, 10°26'N, 84°01'W, 22–24.ii.2012, J.S. Noyes, NHM (Ent) 2012–91”, “HOLOTYPE Eriastichus oasis Hansson” [red printed label], (NHMUK014431058).

#### Additional type material.

***Paratype*** 1♂ “Costa Rica, Cartago, Humo, El Copal, 9°47'N, 83°45'W, 1050–1250 m, 29.ii–6.iii.2008, C. Hansson” (MZLU:7043.2).

#### Diagnosis

**(male).** Head dark brown, scape yellowish brown, pedicel pale brown, flagellum brown; ventral plaque on scape ca. 0.5 × as long as scape (Fig. 42), antenna with dorsobasal setae on F1 0.5 × as long as F1; gaster with lateral tufts of pale and flattened setae on Gt_6_.

#### Description

**(male holotype NHMUK014431058).** Length of body 1.3 mm (paratype 1.4 mm). Head dark brown, scape yellowish brown, pedicel pale brown, flagellum brown, ventral plaque dark brown. Mesoscutum and mesoscutellum black with metallic tinges, dorsellum pale brown, propodeum dark brown. Legs yellowish brown, hind coxa with base dark brown. Gaster dark brown.

***Head*.** Length/width in frontal view 0.8; width/length in dorsal view 2.3; POL/OOL 2.0; WM/MS 1.5; MS/HE 0.5; HE/head length in frontal view 0.6; widths head/mesoscutum 1.2. ***Antenna*.** Pedicel + flagellum length/mesoscutum width 2.1; pedicel + flagellum length/head width 1.9; lengths scape/ventral plaque 2.2; ventral plaque located below the middle of scape; scape length/width 3.1; lengths scape/head (dorsal view) 0.5; scape length/HE 0.8; length/width F1, F2, F3, F4, clava: 3.3, 3.3, 3.3, 3.0, 8.5; length dorsobasal setae on F1/length F1 0.5. ***Mesosoma*.** Length/width 1.5; mesoscutum length/width 0.5; mesoscutellum length/width 0.6; widths SMG/SLG 0.9; enclosed space between SMG length/width 2.0; lengths mesoscutum/mesoscutellum 1.2; lengths mesoscutellum/dorsellum 2.3; lengths mesosoma/gaster 0.7. ***Wings*.**CC length/width 19.0; lengths CC/MV 1.2; lengths MV/ST 2.1; lengths MV/PM 4.1; lengths PM/ST 0.5; submarginal vein with seven setae on dorsal surface (six setae in paratype). ***Gaster*.** With lateral tufts of pale and flattened setae on Gt_6_.

### Eriastichus ononis sp. nov.

Taxon classificationAnimaliaHymenopteraEulophidae

D7A9EE75-E793-5665-8427-620077FBEF55

http://zoobank.org/A538731B-FECE-4287-9566-65DEC5E3F735

Figure 43

#### Type locality.

Costa Rica, Puntarenas, San Vito, Las Cruces, 8°46'N, 82°57'W, 1300 m, 15–16.ii.2006, J.S. Noyes leg.

#### Type specimen.

***Holotype*** male dried and glued to a paper card. Original labels: ”COSTA RICA, Puntarenas, San Vito, Las Cruces, 8°46'N, 82°57'W, 1300 m, 15–16.ii.2006, J.S. Noyes”, “HOLOTYPE Eriastichus ononis Hansson” [red printed label], (NHMUK014431059).

#### Diagnosis

**(male).** Head dark brown, scape yellowish brown, pedicel and flagellum brown; ventral plaque on scape ca. 0.3 × as long as scape (Fig. 43), antenna with dorsobasal setae on F1 0.9 × as long as F1; gaster with lateral tufts of pale and flattened setae on Gt_6_.

#### Description

**(male holotype NHMUK014431059).** Length of body 1.5 mm. Head dark brown, scape yellowish brown, pedicel and flagellum brown, ventral plaque dark brown. Mesoscutum and mesoscutellum black with metallic tinges, dorsellum pale brown, propodeum dark brown. Legs yellowish brown. Gaster dark brown.

***Head*.** Length/width in frontal view 0.8; width/length in dorsal view 2.1; POL/OOL 2.4; WM/MS 1.5; MS/HE 0.5; HE/head length in frontal view 0.6; widths head/mesoscutum 1.2. ***Antenna*.** Pedicel + flagellum length/mesoscutum width 2.5; pedicel + flagellum length/head width 2.0; lengths scape/ventral plaque 3.1; ventral plaque located below the middle of scape; scape length/width 2.8; lengths scape/head (dorsal view) 0.5; scape length/HE 0.9; length/width F1, F2, F3, F4, clava: 3.6, 3.6, 3.6, 3.4, 7.4; length dorsobasal setae on F1/length F1 0.9. ***Mesosoma*.** Length/width 1.7; mesoscutum length/width 0.7; mesoscutellum length/width 0.9; widths SMG/SLG 1.0; enclosed space between SMG length/width 2.7; lengths mesoscutum/mesoscutellum 1.4; lengths mesoscutellum/dorsellum 3.4; lengths mesosoma/gaster 0.7. ***Wings*.**CC length/width 16.0; lengths CC/MV 1.0; lengths MV/ST 2.1; lengths MV/PM 5.0; lengths PM/ST 0.4; submarginal vein with seven setae on dorsal surface. ***Gaster*.** With lateral tufts of pale and flattened setae on Gt_6_.

### Eriastichus orestis sp. nov.

Taxon classificationAnimaliaHymenopteraEulophidae

F25EDA90-A6C1-554F-B9F5-2BD896043257

http://zoobank.org/C962EAC3-7329-4811-A87E-EE829A92C069

Figure 49

#### Type locality.

Costa Rica, Puntarenas, San Vito, Estación Biológica Las Alturas, 1500 m, 8°57'N, 82°50'W, 17–18.ii.2012, J.S. Noyes leg.

#### Type specimen.

***Holotype*** male dried and glued to a paper card. Original labels: ”COSTA RICA, Puntarenas, San Vito, E.B. Las Alturas, 1500 m, 8°57'N, 82°50'W, 17–18.ii.2012, J.S. Noyes, NHM (Ent) 2012–91”, “HOLOTYPE Eriastichus orestis Hansson” [red printed label], (NHMUK014431060).

#### Additional type material.

***Paratypes*** (2♂♂): 1♂ with same label data as holotype (NHMUK014431061); 1♂ “Costa Rica, Cartago, Humo, El Copal, 9°47'N, 83°45'W, 1050–1250 m, 29.ii–6.iii.2008, C. Hansson” (MZLU:7044.2).

#### Diagnosis

**(male).** Head dark brown with part below antennal toruli dark yellowish brown, scape yellowish brown, pedicel and flagellum brown; ventral plaque on scape ca. 0.3 × as long as scape (Fig. 49), antenna with dorsobasal setae on F1 0.9 × as long as F1; gaster with lateral tufts of pale and flattened setae on Gt_6_.

#### Description

**(male holotype NHMUK014431060).** Length of body 1.7 mm (paratypes 1.7–1.8 mm). Head dark brown with part below antennal toruli pale brown, scape yellowish brown, pedicel pale brown, flagellum brown, ventral plaque dark brown. Mesoscutum, mesoscutellum and propodeum dark brown, dorsellum pale brown. Legs yellowish brown. Gaster dark brown.

***Head*.** Length/width in frontal view 0.7; width/length in dorsal view 2.3; POL/OOL 1.8; WM/MS 1.5; MS/HE 0.6; HE/head length in frontal view 0.6; widths head/mesoscutum 1.3. ***Antenna*.** Pedicel + flagellum length/mesoscutum width 2.3; pedicel + flagellum length/head width 1.9; lengths scape/ventral plaque 3.0; ventral plaque located below the middle of scape; scape length/width 3.0; lengths scape/head (dorsal view) 0.5; scape length/HE 0.9; length/width F1, F2, F3, F4, clava: 2.8, 3.0, 3.2, 3.2, 6.8; length dorsobasal setae on F1/length F1 0.9. ***Mesosoma*.** Length/width 1.6; mesoscutum length/width 0.7; mesoscutellum length/width 0.8; widths SMG/SLG 1.1; enclosed space between SMG length/width 2.4; lengths mesoscutum/mesoscutellum 1.5; lengths mesoscutellum/dorsellum 3.3; lengths mesosoma/gaster 0.7. ***Wings*.**CC length/width 16.2; lengths CC/MV 0.7; lengths MV/ST 2.4; lengths MV/PM 4.8; lengths PM/ST 0.5; submarginal vein with six setae on dorsal surface (paratypes with eight setae). ***Gaster*.** With lateral tufts of pale and flattened setae on Gt_6_.

### Eriastichus pallidops sp. nov.

Taxon classificationAnimaliaHymenopteraEulophidae

300DB09D-3843-534E-AE18-F0529DF95309

http://zoobank.org/A4B4B83F-D622-49B8-965F-CECE0B604217

Figures 50 , 59

#### Type locality.

Costa Rica, Cartago, Humo, El Copal, 9°47'N, 83°45'W, 1050–1250 m, 29.ii–6.iii.2008, C. Hansson leg.

#### Type specimen.

***Holotype*** male dried and glued to a paper card. Original labels: ”Costa Rica, Cartago, Humo, El Copal, 9°47'N, 83°45'W, 1050–1250 m, 29.ii–6.iii.2008, C. Hansson”, “HOLOTYPE Eriastichus pallidops Hansson” [red printed label], (MZLU:7045.1).

#### Additional type material.

***Paratypes*** 2♂ with same label data as holotype (MZUCR:01695, NHMUK014431062).

#### Diagnosis

**(male).** Head with lower ½ yellowish brown (Fig. 59); antenna (Fig. 50): scape yellowish brown, ventral plaque on scape 0.7 × as long as scape, antenna with dorsobasal setae on F1 0.4 × as long as F1; gaster with lateral tufts of pale and flattened setae on Gt_5-6_.

#### Description

**(male holotype MZLU:7045.1).** Length of body 1.9 mm (paratypes 1.5–1.7 mm). Head yellowish brown with upper ½ of frons pale brown, vertex and upper ½ of occiput dark brown. Antenna with scape yellowish brown with dorsal margin pale brown, ventral plaque black; pedicel and flagellum brown. Mesoscutum and mesoscutellum black with metallic tinges; dorsellum pale brown; propodeum dark brown. Legs yellowish brown with base of hind coxa and 4^th^ tarsomere dark brown. Gaster dark brown.

***Head*.** Length/width in frontal view 0.7; width/length in dorsal view 2.2; POL/OOL 1.8; WM/MS 1.7; MS/HE 0.5; HE/head length in frontal view 0.6; widths head/mesoscutum 1.1. ***Antenna*.** Pedicel + flagellum length/mesoscutum width 1.5; pedicel + flagellum length/head width 1.4; lengths scape/ventral plaque 1.4; ventral plaque located slightly above the middle of scape; scape length/width 2.8; lengths scape/head (dorsal view) 0.6; scape length/HE 0.9; length/width F1, F2, F3, F4, clava: 2.3, 2.0, 1.8, 1.8, 4.0; length dorsobasal setae on F1/length F1 0.4. ***Mesosoma*.** Length/width 1.6; mesoscutum length/width 0.5; mesoscutellum length/width 0.7; widths SMG/SLG 1.2; enclosed space between SMG length/width 1.8; lengths mesoscutum/mesoscutellum 1.2; lengths mesoscutellum/dorsellum 2.7; lengths mesosoma/gaster 0.8. ***Wings*.**CC length/width 19.3; lengths CC/MV 1.1; lengths MV/ST 2.2; lengths MV/PM 3.7; lengths PM/ST 0.6; submarginal vein with ten setae on dorsal surface (8–9 setae in paratypes). ***Gaster*.** With lateral tufts of pale and flattened setae on Gt_5-6_.

#### Etymology.

Named for the yellowish brown lower face.

### Eriastichus parabilis sp. nov.

Taxon classificationAnimaliaHymenopteraEulophidae

32AD86DB-5A49-506A-B1D5-B9B3886EFDB1

http://zoobank.org/F33852D4-65AF-4B99-9A79-A5C468443274

Figure 46

#### Type locality.

Costa Rica, San José, San Gerardo de Dota, 9°33'N, 83°47'W, 20–21.ii.2013, J.S. Noyes leg.

#### Type specimen.

***Holotype*** male dried and glued to a paper card. Original labels: ”Costa Rica, San José, San Gerardo de Dota, 20–21.ii.2013, J.S. Noyes, NHM (Ent) 2012–91”, “HOLOTYPE Eriastichus parabilis Hansson” [red printed label], (NHMUK014431063).

#### Additional type material.

***Paratype*** 1♂ with same label data as holotype (NHMUK014431064).

#### Diagnosis

**(male).** Head black with lower part of clypeus yellowish brown, scape pale brown, pedicel and flagellum dark brown; ventral plaque on scape ca. 0.4 × as long as scape (Fig. 46), antenna with dorsobasal setae on F1 0.5 × as long as F1; gaster with lateral tufts of pale and flattened setae on Gt_6_.

#### Description

**(male holotype NHMUK014431063).** Length of body 2.3 mm (paratype 2.3 mm). Head black with lower part of clypeus yellowish brown, scape pale brown, pedicel and flagellum dark brown, ventral plaque dark brown. Mesoscutum, mesoscutellum, dorsellum and propodeum black with metallic tinges. Legs with hind coxa black, hind femur pale brown, remaining parts of legs yellowish brown. Wings uniformly but weakly infuscated. Gaster dark brown.

***Head*.** Length/width in frontal view 0.7; width/length in dorsal view 2.3; POL/OOL 1.9; WM/MS 1.5; MS/HE 0.6; HE/head length in frontal view 0.6; widths head/mesoscutum 1.1. ***Antenna*.** Pedicel + flagellum length/mesoscutum width 2.2; pedicel + flagellum length/head width 2.0; lengths scape/ventral plaque 2.8; ventral plaque located below the middle of scape; scape length/width 3.1; lengths scape/head (dorsal view) 0.7; scape length/HE 0.9; length/width F1, F2, F3, F4, clava: 3.1, 3.0, 2.9, 2.9, 6.3; length dorsobasal setae on F1/length F1 0.5. ***Mesosoma*.** Length/width 1.7; mesoscutum length/width 0.7; mesoscutellum length/width 0.8; widths SMG/SLG 1.3; enclosed space between SMG length/width 2.1; lengths mesoscutum/mesoscutellum 1.5; lengths mesoscutellum/dorsellum 3.2; lengths mesosoma/gaster 0.8. ***Wings*.**CC length/width 17.5; lengths CC/MV 1.0; lengths MV/ST 1.9; lengths MV/PM 5.1; lengths PM/ST 0.4; submarginal vein with ten setae on dorsal surface (nine setae in paratype). ***Gaster*.** With lateral tufts of pale and flattened setae on Gt_6_.

### Eriastichus renodis sp. nov.

Taxon classificationAnimaliaHymenopteraEulophidae

6E94145F-8A8E-5FC7-B6B1-A4E84C057421

http://zoobank.org/1BC71A82-5B85-4E61-BD84-27543158E22C

Figure 54

#### Type locality.

Costa Rica, Cartago, Humo, El Copal, 9°47'N, 83°45'W, 1050–1250 m, 29.ii–6.iii.2008, C. Hansson leg.

#### Type specimen.

***Holotype*** male dried and glued to a paper card. Original labels: ”Costa Rica, Cartago, Humo, El Copal, 9°47'N, 83°45'W, 1050–1250 m, 29.ii–6.iii.2008, C. Hansson”, “HOLOTYPE Eriastichus renodis Hansson” [red printed label], (MZLU:7046.1).

#### Diagnosis

**(male).** Head dark brown, scape yellowish brown, pedicel pale brown, flagellum brown; ventral plaque on scape ca. 0.3 × as long as scape (Fig. 54), antenna with dorsobasal setae on F1 0.9 × as long as F1; gaster with lateral tufts of pale and flattened setae on Gt_6_.

#### Description

**(male holotype MZLU:7046.1).** Length of body 1.5 mm. Head dark brown, scape yellowish brown, pedicel pale brown, flagellum brown, ventral plaque dark brown. Mesoscutum and mesoscutellum dark brown with metallic tinges, dorsellum and propodeum dark brown. Legs yellowish brown. Gaster dark brown.

***Head*.** Length/width in frontal view 0.7; width/length in dorsal view 2.2; POL/OOL 2.3; WM/MS 1.3; MS/HE 0.6; HE/head length in frontal view 0.6; widths head/mesoscutum 1.2. ***Antenna*.** Pedicel + flagellum length/mesoscutum width 2.4; pedicel + flagellum length/head width 2.0; lengths scape/ventral plaque 3.1; ventral plaque located in the middle of scape; scape length/width 3.1; lengths scape/head (dorsal view) 0.6; scape length/HE 0.9; length/width F1, F2, F3, F4, clava: 3.2, 3.4, 3.4, 3.4, 7.6; length dorsobasal setae on F1/length F1 0.9. ***Mesosoma*.** Length/width 1.7; mesoscutum length/width 0.6; mesoscutellum length/width 0.9; widths SMG/SLG 1.1; enclosed space between SMG length/width 2.3; lengths mesoscutum/mesoscutellum 1.4; lengths mesoscutellum/dorsellum 2.9; lengths mesosoma/gaster 0.8. ***Wings*.**CC length/width 20.0; lengths CC/MV 0.8; lengths MV/ST 2.3; lengths MV/PM 4.2; lengths PM/ST 0.5; submarginal vein with nine setae on dorsal surface. ***Gaster*.** With lateral tufts of pale and flattened setae on Gt_6_.

### Eriastichus rivalis sp. nov.

Taxon classificationAnimaliaHymenopteraEulophidae

B5EF57BC-6751-5A05-A8BF-80DFD16F3C5B

http://zoobank.org/F78216A7-C07E-4635-A5E5-921D46D5E6AA

Figure 47

#### Type locality.

Costa Rica, San José, San Gerardo de Dota, 9°33'N, 83°47'W, 20–21.ii.2013, J.S. Noyes leg.

#### Type specimen.

***Holotype*** male dried and glued to a paper card. Original labels: ”Costa Rica, San José, San Gerardo de Dota, 20–21.ii.2013, J.S. Noyes, NHM (Ent) 2012–91”, “HOLOTYPE Eriastichus rivalis Hansson” [red printed label], (NHMUK014431065).

#### Diagnosis

**(male).** Head black, scape pale brown, pedicel and flagellum dark brown; ventral plaque on scape ca. 0.4 × as long as scape (Fig. 47), antenna with dorsobasal setae on F1 0.5 × as long as F1; gaster with lateral tufts of pale and flattened setae on Gt_6_.

#### Description

**(male holotype NHMUK014431065).** Length of body 2.0 mm. Head black, scape pale brown, pedicel and flagellum dark brown, ventral plaque dark brown. Mesoscutum, mesoscutellum, dorsellum and propodeum black with metallic tinges. Legs with mid and hind coxae dark brown, hind femur pale brown, remaining parts of legs yellowish brown. Gaster dark brown.

***Head*.** Length/width in frontal view 0.8; width/length in dorsal view 2.3; POL/OOL 2.4; WM/MS 1.4; MS/HE 0.6; HE/head length in frontal view 0.6; widths head/mesoscutum 1.2. ***Antenna*.** Pedicel + flagellum length/mesoscutum width 2.0; pedicel + flagellum length/head width 1.9; lengths scape/ventral plaque 2.8; ventral plaque located below the middle of scape; scape length/width 3.1; lengths scape/head (dorsal view) 0.6; scape length/HE 1.0; length/width F1, F2, F3, F4, clava: 3.7, 3.2, 3.2, 3.2, 7.3; length dorsobasal setae on F1/length F1 0.5. ***Mesosoma*.** Length/width 1.6; mesoscutum length/width 0.6; mesoscutellum length/width 0.7; widths SMG/SLG 1.2; enclosed space between SMG length/width 2,0; lengths mesoscutum/mesoscutellum 1.5; lengths mesoscutellum/dorsellum 2.7; lengths mesosoma/gaster 0.7. ***Wings*.**CC length/width 21.3; lengths CC/MV 1.0; lengths MV/ST 2.2; lengths MV/PM 4.1; lengths PM/ST 0.5; submarginal vein with nine setae on dorsal surface. ***Gaster*.** With lateral tufts of pale and flattened setae on Gt_6_.

### Eriastichus sannionis sp. nov.

Taxon classificationAnimaliaHymenopteraEulophidae

1CAA0B00-211F-5DE3-91D4-7343E9E58442

http://zoobank.org/F30DC6E4-DDEE-4240-B603-BD32C6EA5DE2

Figure 51

#### Type locality.

Costa Rica, Cartago, Humo, El Copal, 9°47'N, 83°45'W, 1050–1250 m, 29.ii–6.iii.2008, C. Hansson leg.

#### Type specimen.

***Holotype*** male dried and glued to a paper card. Original labels: ”Costa Rica, Cartago, Humo, El Copal, 9°47'N, 83°45'W, 1050–1250 m, 29.ii–6.iii.2008, C. Hansson”, “HOLOTYPE Eriastichus sannionis Hansson” [red printed label], (MZLU:7047.1).

#### Additional type material.

***Paratype*** 1♂ with same label data as holotype (NHMUK014431066).

#### Diagnosis

**Figures 51–56.**
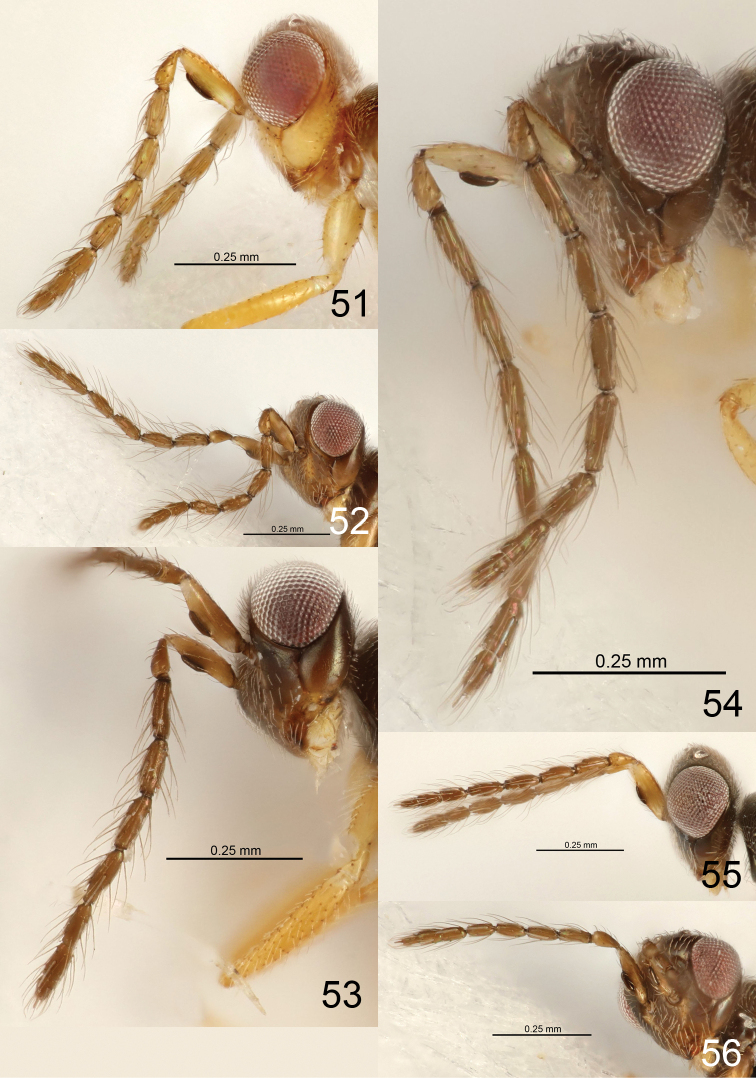
*Eriastichus* spp. head and antenna in lateral view, male holotypes **51***E. sannionis***52***E. scalaris***53***E. taraxis***54***E. renodis***55***E. tendrilis***56***E. velaminis*.

**(male).** Head yellowish brown with vertex and upper ½ of occiput brown, antenna yellowish brown; ventral plaque on scape ca. 0.5 × as long as scape (Fig. 51), antenna with dorsobasal setae on F1 0.6 × as long as F1; gaster with lateral tufts of pale and flattened setae on Gt_5-6_.

#### Description

**(male holotype MZLU:7047.1).** Length of body 1.5 mm (paratype 1.3 mm). Head yellowish brown with vertex and upper ½ of occiput brown, antenna yellowish brown, ventral plaque dark brown. Mesoscutum and propodeum dark brown, mesoscutellum black with metallic tinges, dorsellum pale brown. Legs yellowish brown. Gaster dark brown.

***Head*.** Length/width in frontal view 0.7; width/length in dorsal view 2.2; POL/OOL 2.1; WM/MS 1.5; MS/HE 0.5; HE/head length in frontal view 0.7; widths head/mesoscutum 1.2. ***Antenna*.** Pedicel + flagellum length/mesoscutum width 1.8; pedicel + flagellum length/head width 1.5; lengths scape/ventral plaque 2.2; ventral plaque located above the middle of scape; scape length/width 3.3; lengths scape/head (dorsal view) 0.6; scape length/HE 0.9; length/width F1, F2, F3, F4, clava: 2.3, 2.2, 2.0, 2.0, 5.0; length dorsobasal setae on F1/length F1 0.6. ***Mesosoma*.** Length/width 1.6; mesoscutum length/width 0.6; mesoscutellum length/width 0.8; widths SMG/SLG 1.2; enclosed space between SMG length/width 2.2; lengths mesoscutum/mesoscutellum 1.3; lengths mesoscutellum/dorsellum 3.0; lengths mesosoma/gaster 0.8. ***Wings*.**CC length/width 30.0; lengths CC/MV 1.0; lengths MV/ST 1.9; lengths MV/PM 5.8; lengths PM/ST 0.3; submarginal vein with eight setae on dorsal surface, in both types. ***Gaster*.** With lateral tufts of pale and flattened setae on Gt_5-6_.

### Eriastichus scalaris sp. nov.

Taxon classificationAnimaliaHymenopteraEulophidae

BEA3920A-38F1-5725-A06D-9B1A28A3A772

http://zoobank.org/F4846532-08CB-4CE2-AB52-5FCE0689B574

Figure 52

#### Type locality.

Costa Rica, San José, San Gerardo de Dota, 9°33'N, 83°47'W, 20–21.ii.2013, J.S. Noyes leg.

#### Type specimen.

***Holotype*** male dried and glued to a paper card. Original labels: ”Costa Rica, San José, San Gerardo de Dota, 20–21.ii.2013, J.S. Noyes, NHM (Ent) 2012–91”, “HOLOTYPE Eriastichus scalaris Hansson” [red printed label], (NHMUK014431067).

#### Diagnosis

**(male).** Head dark brown with part below antennaltoruli pale brown, scape pale brown, pedicel and flagellum brown; ventral plaque on scape ca. 0.4 × as long as scape (Fig. 52), antenna with dorsobasal setae on F1 1.0 × as long as F1; gaster with lateral tufts of pale and flattened setae on Gt_5-6_.

#### Description

**(male holotype NHMUK014431067).** Length of body 1.5 mm. Head dark brown with part below antennaltoruli pale brown, scape pale brown, pedicel and flagellum brown, ventral plaque dark brown. Mesoscutum, mesoscutellum and propodeum dark brown, dorsellum pale brown. Legs with fore and mid coxae pale brown, hind coxa and hind femur dark brown, remaining parts of legs yellowish brown. Gaster dark brown.

***Head*.** Length/width in frontal view 0.8; width/length in dorsal view 2.1; POL/OOL 2.0; WM/MS 1.4; MS/HE 0.7; HE/head length in frontal view 0.5; widths head/mesoscutum 1.1. ***Antenna*.** Pedicel + flagellum length/mesoscutum width 2.0; pedicel + flagellum length/head width 1.7; lengths scape/ventral plaque 2.5; ventral plaque located in the middle of scape; scape length/width 3.1; lengths scape/head (dorsal view) 0.5; scape length/HE 1.0; length/width F1, F2, F3, F4, clava: 2.5, 2.3, 2.5, 2.5, 5.8; length dorsobasal setae on F1/length F1 1.0. ***Mesosoma*.** Length/width 1.6; mesoscutum length/width 0.7; mesoscutellum length/width 0.5; widths SMG/SLG 0.9; enclosed space between SMG length/width 2.4; lengths mesoscutum/mesoscutellum 1.5; lengths mesoscutellum/dorsellum 3.0; lengths mesosoma/gaster 0.8. ***Wings*.**CC length/width 16.0; lengths CC/MV 1.0; lengths MV/ST 2.2; lengths MV/PM 7.1; lengths PM/ST 0.3; submarginal vein with seven setae on dorsal surface. ***Gaster*.** With lateral tufts of pale and flattened setae on Gt_5-6_.

### Eriastichus sodalis sp. nov.

Taxon classificationAnimaliaHymenopteraEulophidae

5558142F-F5E8-5758-9E18-B7AB0603CE34

http://zoobank.org/F0A17B16-B048-4891-AF6C-3184ED9B3A21

Figure 48

#### Type locality.

Costa Rica, San José, San Gerardo de Dota, 9°33'N, 83°47'W, 20–21.ii.2013, J.S. Noyes leg.

#### Type specimen.

***Holotype*** male dried and glued to a paper card. Original labels: ”Costa Rica, San José, San Gerardo de Dota, 20–21.ii.2013, J.S. Noyes, NHM (Ent) 2012–91”, “HOLOTYPE Eriastichus sodalis Hansson” [red printed label], (NHMUK014431068).

#### Diagnosis

**(male).** Head dark brown with metallic tinges, scape pale brown, pedicel and flagellum dark brown; ventral plaque on scape ca. 0.3 × as long as scape (Fig. 48), antenna with dorsobasal setae on F1 0.8 × as long as F1; gaster with lateral tufts of pale and flattened setae on Gt_6_.

#### Description

**(male holotype NHMUK014431068).** Length of body 1.9 mm. Head dark brown with metallic tinges, scape pale brown, pedicel and flagellum dark brown, ventral plaque dark brown. Mesoscutum and mesoscutellum black with metallic tinges, dorsellum dark brown, propodeum black. Legs with mid coxa and hind femur pale brown, hind coxa dark brown, remaining parts of legs yellowish brown. Gaster dark brown.

***Head*.** Length/width in frontal view 0.8; width/length in dorsal view 2.2; POL/OOL 1.6; WM/MS 1.6; MS/HE 0.5; HE/head length in frontal view 0.6; widths head/mesoscutum 1.1. ***Antenna*.** Pedicel + flagellum length/mesoscutum width 2.0; pedicel + flagellum length/head width 1.9; lengths scape/ventral plaque 3.0; ventral plaque located in the middle of scape; scape length/width 2.9; lengths scape/head (dorsal view) 0.5; scape length/HE 0.9; length/width F1, F2, F3, F4, clava: 2.9, 2.6, 2.6, 2.4, 5.7; length dorsobasal setae on F1/length F1 0.8. ***Mesosoma*.** Length/width 1.7; mesoscutum length/width 0.7; mesoscutellum length/width 0.8; widths SMG/SLG 1.1; enclosed space between SMG length/width 2.2; lengths mesoscutum/mesoscutellum 1.5; lengths mesoscutellum/dorsellum 2.8; lengths mesosoma/gaster 0.7. ***Wings*.**CC length/width 19.3; lengths CC/MV 1.0; lengths MV/ST 2.2; lengths MV/PM 5.3; lengths PM/ST 0.4; submarginal vein with six setae on dorsal surface. ***Gaster*.** With lateral tufts of pale and flattened setae on Gt_6_.

### Eriastichus taraxis sp. nov.

Taxon classificationAnimaliaHymenopteraEulophidae

142E9D0E-4ECC-5A83-B7E2-C6A71739C47C

http://zoobank.org/F6E6E4E6-E788-4C49-BA77-2965F2B87E70

Figure 53

#### Type locality.

Costa Rica, Puntarenas, Estación Biológica Monteverde, 10°20'N, 84°49'W, 1540 m, 26.ii.2007, J.S. Noyes leg.

#### Type specimen.

***Holotype*** male dried and glued to a paper card. Original labels: ”Costa Rica, Puntarenas, E.B. Monteverde, 10°20'N, 84°49'W, 1540 m, 26.ii.2007, J.S. Noyes”, “HOLOTYPE Eriastichus taraxis Hansson” [red printed label], (NHMUK014431069).

#### Diagnosis

**(male).** Head dark brown, scape yellowish brown, pedicel pale brown, flagellum brown; ventral plaque on scape ca. 0.4 × as long as scape (Fig. 53), antenna with dorsobasal setae on F1 0.8 × as long as F1; gaster with lateral tufts of pale and flattened setae on Gt_6_.

#### Description

**(male holotype NHMUK014431069).** Length of body 1.6 mm. Head dark brown, scape yellowish brown, pedicel pale brown, flagellum brown, ventral plaque dark brown. Mesoscutum and mesoscutellum black with metallic tinges, dorsellum and propodeum dark brown. Legs yellowish brown, hind coxa with base dark brown. Gaster dark brown.

***Head*.** Length/width in frontal view 0.8; width/length in dorsal view 2.1; POL/OOL 2.1; WM/MS 1.5; MS/HE 0.5; HE/head length in frontal view 0.6; widths head/mesoscutum 1.1. ***Antenna*.** Pedicel + flagellum length/mesoscutum width 2.1; pedicel + flagellum length/head width 1.9; lengths scape/ventral plaque 2.7; ventral plaque located in the middle of scape; scape length/width 3.1; lengths scape/head (dorsal view) 0.5; scape length/HE 0.8; length/width F1, F2, F3, F4, clava: 3.0, 3.0, 3.0, 2.8, 6.3; length dorsobasal setae on F1/length F1 0.8. ***Mesosoma*.** Length/width 1.6; mesoscutum length/width 0.7; mesoscutellum length/width 0.8; widths SMG/SLG 1.1; enclosed space between SMG length/width 2.3; lengths mesoscutum/mesoscutellum 1.6; lengths mesoscutellum/dorsellum 2.8; lengths mesosoma/gaster 0.7. ***Wings*.**CC length/width 22.5; lengths CC/MV 1.0; lengths MV/ST 2.0; lengths MV/PM 6.5; lengths PM/ST 0.3; submarginal vein with seven setae on dorsal surface. ***Gaster*.** With lateral tufts of pale and flattened setae on Gt_6_.

### Eriastichus tendrilis sp. nov.

Taxon classificationAnimaliaHymenopteraEulophidae

EA04FD74-04A1-5357-9E23-5E0660BF352A

http://zoobank.org/1EF86DE6-2C33-48A8-9555-08C10059AC76

Figure 55

#### Type locality.

Costa Rica, Puntarenas, Estación Biológica Monteverde, 10°20'N, 84°49'W, 1540 m, 18–25.ii.2004, C. Hansson leg.

#### Type specimen.

***Holotype*** male dried and glued to a paper card. Original labels: ”COSTA RICA, Puntarenas, E.B Monteverde, 10°20'N, 84°49'W, 1540 m, 18–25.ii.2004, C. Hansson”, “HOLOTYPE Eriastichus tendrilis Hansson” [red printed label], (MZLU:7048.1).

#### Additional type material.

***Paratypes*** (5♂♂): 2♂♂ with same label data as holotype (MZLU:7048.2, MZUCR:01696); 2♂♂ from same locality as holotype but collected 26.ii.2007 (NHMUK014431070, NHMUK014431071); 1♂ “Costa Rica, San José, San Gerardo de Dota, El Manantial, 18–20.ii.2010, J.S. Noyes, NHM (Ent) 2010–21” (NHMUK014431072).

#### Diagnosis

**(male).** Head black with metallic tinges, scape yellowish brown, pedicel and flagellum brown; ventral plaque on scape ca. 0.3 × as long as scape (Fig. 55), antenna with dorsobasal setae on F1 0.6 × as long as F1; gaster with lateral tufts of pale and flattened setae on Gt_6_.

#### Description

**(male holotype MZLU:7048.1).** Length of body 1.5 mm (paratypes 1.4–1.7 mm). Head black with metallic tinges, scape yellowish brown, pedicel and flagellum brown, ventral plaque dark brown. Mesoscutum and mesoscutellum black with metallic tinges, dorsellum and propodeum black. Legs with fore and mid coxae yellowish brown with base brown, hind coxa black, hind femur pale brown, remaining parts of legs yellowish brown. Gaster dark brown.

***Head*.** Length/width in frontal view 0.7; width/length in dorsal view 2.5; POL/OOL 1.8; WM/MS 1.6; MS/HE 0.6; HE/head length in frontal view 0.6; widths head/mesoscutum 1.1. ***Antenna*.** Pedicel + flagellum length/mesoscutum width 1.5; pedicel + flagellum length/head width 1.5; lengths scape/ventral plaque 3.0; ventral plaque located in the middle of scape; scape length/width 3.0; lengths scape/head (dorsal view) 0.6; scape length/HE 0.9; length/width F1, F2, F3, F4, clava: 2.7, 2.7, 2.5, 2.5, 5.5; length dorsobasal setae on F1/length F1 0.6. ***Mesosoma*.** Length/width 1.5; mesoscutum length/width 0.7; mesoscutellum length/width 0.8; widths SMG/SLG 1.0; enclosed space between SMG length/width 2.0; lengths mesoscutum/mesoscutellum 1.6; lengths mesoscutellum/dorsellum 3.0; lengths mesosoma/gaster 1.0. ***Wings*.**CC length/width 18.0; lengths CC/MV 1.0; lengths MV/ST 2.1; lengths MV/PM 6.5; lengths PM/ST 0.3; submarginal vein with six setae on dorsal surface (7–8 setae in paratypes). ***Gaster*.** With lateral tufts of pale and flattened setae on Gt_6_.

### Eriastichus tonioazofeifai sp. nov.

Taxon classificationAnimaliaHymenopteraEulophidae

79DF4310-3C66-5BA9-82DD-6E51D15288B8

http://zoobank.org/4E666BE9-F215-4776-AA27-1E865C9F7A12

Figure 57

#### Type locality.

Costa Rica, Heredia, Estación Biológica La Selva, 100–200 m, 10°26'N, 84°01'W, 30–31.iii.2002, swept, J.A. Azofeifa leg.

#### Type specimen.

***Holotype*** male dried and glued to a paper card. Original labels: ”COSTA RICA, Heredia, E.B. La Selva, 100–200 m, 10°26'N, 84°01'W, 30–31.iii.2002, swept, J.A. Azofeifa,”, “HOLOTYPE Eriastichus tonioazofeifai Hansson” [red printed label], (MZLU:7049.1).

#### Diagnosis

**Figures 57–60.**
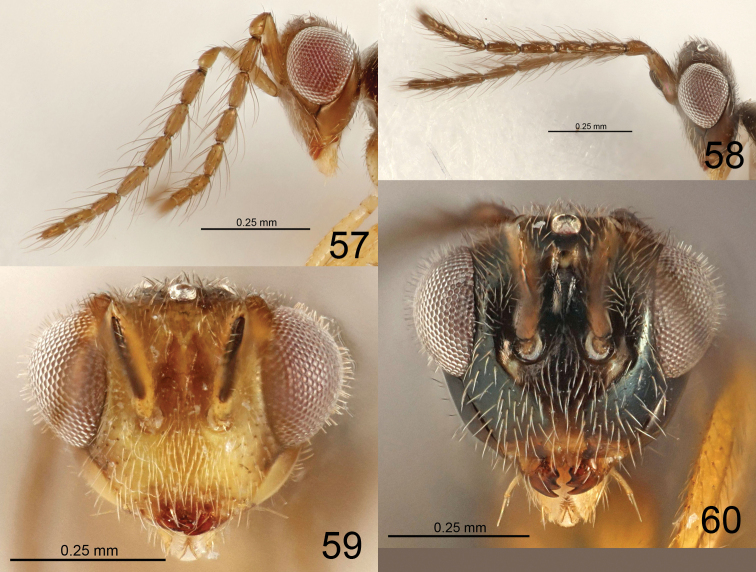
*Eriastichus* spp. **57, 58** head and antenna in lateral view, male holotypes: **57***E. tonioazofeifai***58***E. vestis*; **59, 60** head in frontal view, male holotypes: **59***E. pallidops***60***E. denotatis*.

**(male).** Head dark brown with part below antennal toruli yellowish brown; antenna (Fig. 57): scape pale brown, ventral plaque on scape ca. 0.1 × as long as scape, antenna with dorsobasal setae on F1 1.1 × as long as F1; gaster with lateral tufts of pale and flattened setae on Gt_6_.

#### Description

**(male holotype MZLU:7049.1).** Length of body 1.3 mm. Head dark brown with part below antennal toruli yellowish brown. Antenna with scape pale brown, ventral plaque dark brown, pedicel and flagellum brown. Mesoscutum and mesoscutellum dark brown with metallic tinges; dorsellum pale brown; propodeum dark brown. Legs yellowish brown with hind coxa and hind femur pale brown. Wings ±hyaline. Gaster dark brown.

***Head*.** Length/width in frontal view 0.7; width/length in dorsal view 2.3; POL/OOL 2.5; WM/MS 1.5; MS/HE 0.5; HE/head length in frontal view 0.6; widths head/mesoscutum 1.2. ***Antenna*.** Pedicel + flagellum length/mesoscutum width 2.0; pedicel + flagellum length/head width 1.6; lengths scape/ventral plaque 8.0; ventral plaque located in the middle of scape; scape length/width 5.8; lengths scape/head (dorsal view) 0.8; scape length/HE 1.3; length/width F1, F2, F3, F4, clava: 2.0, 2.0, 2.0, 2.2, 5.3; length dorsobasal setae on F1/length F1 1.1. ***Mesosoma*.** Length/width 1.5; mesoscutum length/width 0.6; mesoscutellum length/width 0.7; widths SMG/SLG 1.1; enclosed space between SMG length/width 2.0; lengths mesoscutum/mesoscutellum 1.4; lengths mesoscutellum/dorsellum 2.9; lengths mesosoma/gaster 0.7. ***Wings*.**CC length/width 21.0; lengths CC/MV 1.3; lengths MV/ST 1.9; lengths MV/PM 3.6; lengths PM/ST 0.5; submarginal vein with six setae on dorsal surface. ***Gaster*.** With lateral tufts of pale and flattened setae on Gt_6_.

#### Etymology.

Named after collector, Antonio (Tonio) Azofeifa, parataxonomist in Costa Rica.

### Eriastichus velaminis sp. nov.

Taxon classificationAnimaliaHymenopteraEulophidae

D8D2FA2B-693E-533D-B2BB-A0A9976604CB

http://zoobank.org/1263C4A3-410D-4C60-A610-8F8CF591E7A9

Figure 56

#### Type locality.

Costa Rica, San José, San Gerardo de Dota, 9°33'N, 83°47'W, 20–21.ii.2013, J.S. Noyes leg.

#### Type specimen.

***Holotype*** male dried and glued to a paper card. Original labels: ”Costa Rica, San José, San Gerardo de Dota, 20–21.ii.2013, J.S. Noyes, NHM (Ent) 2012–91”, “HOLOTYPE Eriastichus velaminis Hansson” [red printed label], (NHMUK014431073).

#### Diagnosis

**(male).** Head dark brown with metallic tinges, scape and pedicel pale brown, flagellum brown; ventral plaque on scape ca. 0.4 × as long as scape (Fig. 56), antenna with dorsobasal setae on F1 0.8 × as long as F1; gaster with lateral tufts of pale and flattened setae on Gt_6_.

#### Description

**(male holotype NHMUK014431073).** Length of body 1.2 mm. Head dark brown with metallic tinges, scape and pedicel pale brown, flagellum brown, ventral plaque dark brown. Mesoscutum and mesoscutellum black with metallic tinges, dorsellum and propodeum dark brown. Legs with coxae and femora on fore and mid legs pale brown, hind coxa and hind femur dark brown, remaining parts of legs yellowish brown. Gaster dark brown.

***Head*.** Length/width in frontal view 0.8; width/length in dorsal view 2.1; POL/OOL 1.9; WM/MS 1.4; MS/HE 0.6; HE/head length in frontal view 0.5; widths head/mesoscutum 1.1. ***Antenna*.** Pedicel + flagellum length/mesoscutum width 1.8; pedicel + flagellum length/head width 1.5; lengths scape/ventral plaque 2.8; ventral plaque located below the middle of scape; scape length/width 3.1; lengths scape/head (dorsal view) 0.5; scape length/HE 1.0; length/width F1, F2, F3, F4, clava: 2.4, 2.4, 2.2, 2.0, 5.4; length dorsobasal setae on F1/length F1 0.8. ***Mesosoma*.** Length/width 1.5; mesoscutum length/width 0.6; mesoscutellum length/width 0.8; widths SMG/SLG 1.1; enclosed space between SMG length/width 2.0; lengths mesoscutum/mesoscutellum 1.5; lengths mesoscutellum/dorsellum 2.5; lengths mesosoma/gaster 0.8. ***Wings*.**CC length/width 22.0; lengths CC/MV 1.0; lengths MV/ST 2.1; lengths MV/PM 4.7; lengths PM/ST 0.5; submarginal vein with five setae on dorsal surface. ***Gaster*.** With lateral tufts of pale and flattened setae on Gt_6_.

### Eriastichus vestis sp. nov.

Taxon classificationAnimaliaHymenopteraEulophidae

EC283969-F287-567F-AB77-CAF4C24773BD

http://zoobank.org/59E0A806-AAE9-4C16-B373-23BBE4FE3BDC

Figure 58

#### Type locality.

Costa Rica, Puntarenas, San Vito, Estación Biológica Las Alturas, 1500 m, 8°57'N, 82°50'W, 17–18.ii.2012, J.S. Noyes leg.

#### Type specimen.

***Holotype*** male dried and glued to a paper card. Original labels: ”COSTA RICA, Puntarenas, San Vito, E.B. Las Alturas, 1500 m, 8°57'N, 82°50'W, 17–18.ii.2012, J.S. Noyes, NHM (Ent) 2012–91”, “HOLOTYPE Eriastichus vestis Hansson” [red printed label], (NHMUK014431074).

#### Diagnosis

**(male).** Head dark brown, scape and pedicel pale brown, flagellum brown; ventral plaque on scape ca. 0.3 × as long as scape (Fig. 58), antenna with dorsobasal setae on F1 0.9 × as long as F1; gaster with lateral tufts of pale and flattened setae on Gt_6_.

#### Description

**(male holotype NHMUK014431074).** Length of body 1.5 mm. Head dark brown, scape and pedicel pale brown, flagellum brown, ventral plaque dark brown. Mesoscutum, mesoscutellum and propodeum black with metallic tinges, dorsellum dark brown. Legs with coxae and femora dark brown, remaining parts of legs yellowish brown. Gaster dark brown.

***Head*.** Length/width in frontal view 0.8; width/length in dorsal view 2.4; POL/OOL 1.9; WM/MS 1.5; MS/HE 0.6; HE/head length in frontal view 0.5; widths head/mesoscutum 1.0. ***Antenna*.** Pedicel + flagellum length/mesoscutum width 1.8; pedicel + flagellum length/head width 1.8; lengths scape/ventral plaque 2.9; ventral plaque located above the middle of scape; scape length/width 2.9; lengths scape/head (dorsal view) 0.5; scape length/HE 1.0; length/width F1, F2, F3, F4, clava: 3.0, 3.2, 3.0, 3.0, 7.4; length dorsobasal setae on F1/length F1 0.9. ***Mesosoma*.** Length/width 1.5; mesoscutum length/width 0.6; mesoscutellum length/width 0.7; widths SMG/SLG 1.5; enclosed space between SMG length/width 1.7; lengths mesoscutum/mesoscutellum 1.4; lengths mesoscutellum/dorsellum 2.5; lengths mesosoma/gaster 0.9. ***Wings*.**CC length/width 16.0; lengths CC/MV 1.0; lengths MV/ST 2.0; lengths MV/PM 5.3; lengths PM/ST 0.4; submarginal vein with five setae on dorsal surface. ***Gaster*.** With lateral tufts of pale and flattened setae on Gt_6_.

## Discussion

The original description of *Eriastichus* was based on relatively few specimens and was a morphologically distinct genus. With the addition here of some unique features on the gaster of both sexes, the inflated pleural membrane between Gt_1-4_ and Gs_1-4,_ and the tuft of pale and flattened setae laterally on Gt_4-6,_ as well as the features given by La Salle in the original description, *Eriastichus* forms a morphologically well-defined and quite remarkable genus of the Tetrastichinae.

Species included here can only be separated through morphological characters in males, mainly on the antennae, as demonstrated in the identification key. Apart from the antennae, males of the different species are very similar, with the exception of variation in colour on the head and legs in some of the species. The antennae in females show very little variation, which is also the case for other morphological features. Thus, females cannot be separated to species, and they cannot be linked to conspecific males using morphology. Molecular data will be essential for the association of sexes of conspecific specimens, and for the identification of females.

The three species included in the original description were based mainly on females, and with relatively few specimens. Two of the species had the mesoscutellum with parts lateral to submedian grooves strongly setose, a character state not present in any of the new species described here. The third species included males from Mexico, separated in the key from species described here.

Most of the newly described species are represented by single specimens, thus eliminating the possibility of describing morphological variation within species. However, in species with two or more specimens, frequently from different localities and different collecting events, the antennal characters used to separate species show very little or no variation.

## Supplementary Material

XML Treatment for Eriastichus

XML Treatment for Eriastichus acribis

XML Treatment for Eriastichus aphritis

XML Treatment for Eriastichus cigdemae

XML Treatment for Eriastichus cluridis

XML Treatment for Eriastichus coelotis

XML Treatment for Eriastichus colenis

XML Treatment for Eriastichus copalensis

XML Treatment for Eriastichus daptilis

XML Treatment for Eriastichus decoris

XML Treatment for Eriastichus denotatis

XML Treatment for Eriastichus derilis

XML Treatment for Eriastichus diadrys

XML Treatment for Eriastichus dotaensis

XML Treatment for Eriastichus drupis

XML Treatment for Eriastichus ebulis

XML Treatment for Eriastichus egrestis

XML Treatment for Eriastichus eleagnis

XML Treatment for Eriastichus ellipsis

XML Treatment for Eriastichus eminis

XML Treatment for Eriastichus facilis

XML Treatment for Eriastichus fenestris

XML Treatment for Eriastichus follis

XML Treatment for Eriastichus galeatis

XML Treatment for Eriastichus geratis

XML Treatment for Eriastichus glanis

XML Treatment for Eriastichus hilaris

XML Treatment for Eriastichus johnlasallei

XML Treatment for Eriastichus johnnoyesi

XML Treatment for Eriastichus maniatis

XML Treatment for Eriastichus masneri

XML Treatment for Eriastichus nakos

XML Treatment for Eriastichus nebulis

XML Treatment for Eriastichus neonis

XML Treatment for Eriastichus nexilis

XML Treatment for Eriastichus novalis

XML Treatment for Eriastichus nugalis

XML Treatment for Eriastichus oasis

XML Treatment for Eriastichus ononis

XML Treatment for Eriastichus orestis

XML Treatment for Eriastichus pallidops

XML Treatment for Eriastichus parabilis

XML Treatment for Eriastichus renodis

XML Treatment for Eriastichus rivalis

XML Treatment for Eriastichus sannionis

XML Treatment for Eriastichus scalaris

XML Treatment for Eriastichus sodalis

XML Treatment for Eriastichus taraxis

XML Treatment for Eriastichus tendrilis

XML Treatment for Eriastichus tonioazofeifai

XML Treatment for Eriastichus velaminis

XML Treatment for Eriastichus vestis
